# Fine-Tuning of the Endoplasmic Reticulum Stress Response Mechanism Plays a Key Role in Cellular Survival—A Mathematical Study

**DOI:** 10.3390/ijms262210961

**Published:** 2025-11-12

**Authors:** Marianna Holczer, Margita Márton, Ibolya Stiller, Beáta Lizák, Gábor Bánhegyi, Orsolya Kapuy

**Affiliations:** Department of Molecular Biology, Institute of Biochemistry and Molecular Biology, Semmelweis University, 1094 Budapest, Hungary; holczer.marianna@semmelweis.hu (M.H.); marton.margitta@semmelweis.hu (M.M.); stiller.ibolya@semmelweis.hu (I.S.); lizak.beata@semmelweis.hu (B.L.); banhegyi.gabor@med.semmelweis-univ.hu (G.B.)

**Keywords:** endoplasmic reticulum stress, feedback loops, autophagy, apoptosis, systems biology

## Abstract

Proper functioning of the endoplasmic reticulum (ER) plays a key role in maintaining the internal homeostasis of the cell. A common feature of many common diseases (such as diabetes and inflammatory bowel diseases) is the induction of ER stress in cells. While some ER stress is beneficial for cellular survival, high levels of stress can lead to cell death. For this reason, many studies are focused on understanding the exact mechanism of the ER stress response. There are a variety of well-established stressors on the market that can be used to induce ER stress under laboratory conditions (i.e., thapsigargin and tunicamycin). However, new scientific results suggest that these ER stressors act very differently on the stress response mechanism and, therefore, cannot always be used reliably. By using various mathematical methods, our systems biology approach presented here seeks to answer how the well-known ER stressors affect the dynamic characteristic of the control network, specifically highlighting how we can delay the negative impact of ER stress. Furthermore, using mathematical models, we make suggestions on which ER stressors may be useful in which therapeutic treatment.

## 1. Introduction

The endoplasmic reticulum (ER) is a membranous organelle found in all eukaryotic cells, whose functions include synthesis, post-translational modification, and promotion of the native structure of intracellular proteins. It has an important role in a wide range of cellular processes, such as Ca^2+^ storage and lipid biosynthesis, and it is also involved in several signaling mechanisms (i.e., UPR, ER-overload response, and the SREBP (Sterol Regulatory Element-Binding Protein) pathway) [1,2,3]. Therefore, the proper functioning of ER is crucial in maintaining internal homeostasis, processing appropriate signals and stimuli and generating responses to them [3,4].

If misfolded proteins accumulate in the lumen of the endoplasmic reticulum for any reason (e.g., nutrient deficiency, imbalance in Ca^2+^ metabolism, toxin exposure, oxidative stress, viral infection, and mutant protein), the organelle homeostasis is disturbed, thereby inducing several processes, which is called the ER stress response. This mechanism primarily promotes the induction of the unfolded protein response (UPR) signaling pathway [5]. The main function of UPR is to restore the internal homeostasis of ER through various signal transduction pathways. Thus, it enhances the re-folding or degradation of proteins through the induction of various chaperones and enzymes, and it also reduces the protein load on the organelle by stopping de novo protein synthesis [1]. Meanwhile, autophagy-dependent self-digestion is triggered to help restore ER and cellular homeostasis by degrading damaged or unnecessary components. However, in the case of prolonged or severe ER stress, UPR turns on various cell death processes (such as necrosis or apoptosis). Apoptotic cell death ensures that damaged proteins are degraded in a controlled manner and thus avoids the activation of inflammatory processes [5,6,7,8,9].

Numerous human studies have already shown that inadequate ER stress response can lead to the development of various chronic diseases such as diabetes mellitus, neurodegenerative disorders (Parkinson disease, Alzheimer disease, and Huntington’s disease), fatty liver disease, inflammatory bowel disease, and cardiovascular diseases (heart failure, atherosclerosis, plaque rupture, and ischemic heart disease) through the induction of apoptosis and inflammation [10,11]. Therefore, the induction of ER stress under laboratory conditions with various chemicals and the study and understanding of the stress mechanism may have very important medical implications. Tunicamycin (TM) is a natural antibiotic that can disrupt protein folding through inhibiting N-linked glycosylation, resulting in activation of the ER stress response mechanism [12,13]. Thapsigargin (TG) can be found in *Thapsia garganica* L., and its main effect is the inhibition of SERCA (sarcoplasmic Ca^2+^-ATPase), thus causing increased Ca^2+^ concentration in the cytoplasm [14]. The imbalance of Ca^2+^ homeostasis leads to the inactivation of Ca^2+^-dependent chaperones in the ER, resulting in the accumulation of unfolded proteins [15]. Dithiothreitol (DTT), an organosulfur compound, reduces disulfide bonds between cysteine residues, leading to the disruption of redox homeostasis of the ER lumen, protein denaturation, and accumulation of misfolded proteins [15].

Recently, we built up a simple mathematical model of cellular stress response mechanism focusing on the decision-making process between autophagy and apoptosis [16]. On that model, the two processes were controlled simultaneously via a crosstalk element. The key components were grouped into three well-defined groups called the crosstalk element, autophagy, and apoptosis inducer, respectively. By using the systems’ biological methods, we supposed that the crosstalk element had a negative effect on both mechanisms; meanwhile, a double-negative feedback loop was presented between the autophagy and apoptosis inducers. In addition, the inactive form of the autophagy inducer was able to enhance the activation of apoptosis, generating an extra positive feedback loop in the control network. We claimed that these feedback loops guaranteed that at a given stress level, only one mechanism was activated. Namely, a low level of cellular stress enhanced a rapid activation of autophagy-dependent cellular survival; however, an excessive level of stress resulted in a transient autophagy, followed by apoptotic cell death. This model described the dynamic behavior of the stress response in general but did not go into detail about the different effects of different cellular stress events [16].

Here, we focus on the ER stress response mechanism in terms of the addition of various well-known artificial ER stressors (such as TM, TG, and DTT). We assume that an appropriate concentration of TM can be reliably used to induce autophagy-dependent survival, while treatments with DTT or TG have more drastic effects on cell viability. Here, we claim that the amount of these ER stressors is additive and might be fatal for the cell. Therefore, we suppose that the usage of any of these ER stressors requires a very precise control in the case of medical treatments.

## 2. Results

### 2.1. ER Stress Response Mechanism Involves the Proper Balance of UPR, Autophagy, and Apoptosis Pathways

**Figure 1:**
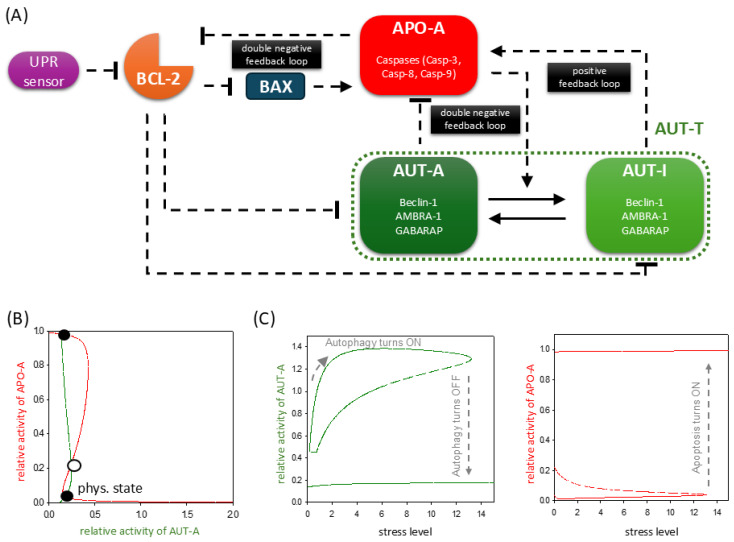
ER stress response mechanism involves the proper balance of UPR, autophagy, and apoptosis pathways. (**A**) The wiring diagram of the control network in relation to ER stress. The UPR sensor, BCL-2, BAX, autophagy inducer (AUT-A), and apoptosis inducer (APO-A) are grouped together in isolated purple, orange, blue, green, and red boxes, respectively. AUTA-T refers to AUTA-A and AUT-I together. The active forms of the molecules are grouped in dark-colored boxes, while a light-colored box denotes the inactive form. Solid arrows represent biochemical reactions, and dashed lines show how the molecules can influence each other. Blocked end lines denote inhibition. (**B**) Phase plane diagrams are plotted under physiological conditions. The balance curves of AUT-A (green) and APO-A (red) are plotted. The phase plane is shown for stress = 0. Intersection of nullclines represents the stable (black dots) and unstable (unfilled circle) steady states. (**C**) The signal response curves of (panel left) AUT-A and (panel right) APO-A are shown with respect to the increasing stress level. Solid lines denote stable states, while dashed lines denote the unstable state.

Our previous simple mathematical model focusing on the regulatory connections between autophagy and apoptosis under cellular stress has been modified to describe directly the ER stress response mechanism with reasonable accuracy (see Figure 1A). In this wiring diagram, a signaling molecule, called UPR, senses ER stress, which has a negative effect on BCL-2 [17], the anti-apoptotic member of the BCL-2 family [18]. According to the literature, BCL-2 inhibits both apoptosis and autophagy through the regulation of the key molecules of the two processes, Caspase-3 [18,19] and Beclin-1 [20], respectively. The negative effect of BCL-2 on Caspase-3 is indirect through inhibition of the formation of the BAK-BAX pore in the mitochondrial outer membrane, thus preventing the activation of apoptosome [21,22]. BCL-2 inhibits another autophagy, promoting proteins, such as AMBRA-1 [23,24,25] and GABARAP [26], strongly suggesting that it has a negative effect on other activators as well that have not yet been experimentally verified. Therefore, we suppose that AUT-A (the autophagy inducer) and APO-A (the apoptosis inducer) group together proteins with similar functional conditions: both groups are inhibited by BCL-2 but induce autophagy or apoptosis, respectively. Since both AUT-A and APO-A are blocked by BCL-2, the ER-stress-dependent diminishment of BCL-2 turns on both the cell survival and cell death mechanism, but which will be active is determined by other down-stream effects.

There is a well-known double-negative feedback loop between AUT-A and APO-A, establishing a mutual antagonism between the two processes (Figure 1A). Additionally, the inactive form of the autophagy inducer (called AUT-I) generated by the caspase-dependent cleavage [27,28,29,30] is able to enhance apoptosis via APO-A induction building in an extra positive feedback loop in the control network. Corresponding to our previous model, we assume that the active caspases can cleave and, therefore, inhibit BCL-2 [31,32], supposing here another double-negative feedback loop between BCL-2 and APO-A. All regulatory connections included in our simple model are verified by already published, experimental scientific results. In addition, through a thorough review of the experimental literature, we have collected the possible proteins that could be included in the APO-A, AUT-A, and AUT-I groups, respectively (for all the collected data, see Figure 1A and Table S1).

To explore the dynamical characteristics of this simple mathematical model, the network diagram is translated into a non-linear differential equation system (for details, see Section 4 and Supplementary Information). First, balance curves are generated, where our model is simplified to a pair of differential equation for AUT-A/dt and APO-A/dt, respectively. We assume that all the other components are in a steady state; meanwhile, the balance curves for AUT-A (the green curve) and APO-A (the red curve) are plotted in an x–y coordinated system (Figure 1B). Along the balance curve, the rate of the active form of the given component is exactly balanced by its rate of the inactive form. The intersections between two balance curves are called equilibrium points; here, the system has steady-state solutions, representing the possible, observable physiological states of the regulatory system. Due to the bistability in the control network, the balance curves have two stable intersections (black dots in Figure 1B) separated by an unstable one (white dot in Figure 1B). Which stable state the system is in always depends on the initial conditions. Under physiological conditions, the system occupies that stable state where APO-A is inactive and AUT-A has some insignificant activity (see label “phys. state” in Figure 1B). This is consistent with the fact that there can be some basal autophagy in the cell, while apoptosis is completely inhibited.

In the case when AUT-A or APO-A is plotted as a function of the ER stress level (the so-called bifurcation parameter), the signal response curves suggest a non-continuous bistable behavior of the system (Figure 1C). Namely, two stable states are separated (see solid lines in Figure 1C) by an unstable state (see dashed lines in Figure 1C), which cannot be reached physically by the control system, thereby resulting in a discontinuous switch between the two stable states. As the stress level increases, BCL-2 releases both AUT-A and APO-A, but in the battle between the two mechanisms, AUT-A wins first. At a low level of ER stress, the active form of AUT-A has a hyperbolic activation followed by a Z-shaped curve as the stress level is increasing (see the grey dashed arrow in Figure 1C, panel left). Meanwhile, the response curve of APO-A is S-shaped upon ER stress (Figure 1C, panel right), confirming that in the first stable state, AUT-A is fully activated and APO-A remains inactive. This corresponds to the state where the cell tries to save itself by autophagy-dependent self-digestion during tolerable ER stress. APO-A activity occupies a lower stable steady state until the stress level reaches the limit point. At this critical threshold, the APO-A activity switches to the higher stable steady state; meanwhile, AUT-A is quickly inhibited, suggesting the induction of cell death processes (see the grey dashed arrows in Figure 1C). The signal response curves also indicate that both autophagy and apoptosis cannot coexist together during different stress levels.

Our model suggests that the simple wiring network of the autophagy and apoptosis inducers generates a bistable system with a sigmoid activation of autophagy, followed by the irreversible induction of apoptosis; however, under physiological conditions, both mechanisms are downregulated.

### 2.2. TM-Induced ER Stress Acts Indirectly on Autophagy and Apoptosis Pathways via UPR

ER stress can be induced under laboratory conditions by different well-known stressors, such as tunicamycin (TM), thapsigargin (TG), or dithiothreitol (DTT) [15]. As different artificial stressors do not interfere equally with the functioning of the regulatory network, we wondered how these drugs can affect the stress response. However, the extent to which the dynamic behavior of the regulatory system is influenced by the stressor with which the cells are treated has not been thoroughly investigated yet. By exploring the literature and studying previously published results, we have collected data on the effects of these three different ER stressors on the ER stress response (see Table S2). These results suggest that treatment with TM, DTT, and TG has differences in terms of the induction of autophagy and apoptosis; therefore, their effects on cell viability might also be different.

**Figure 2:**
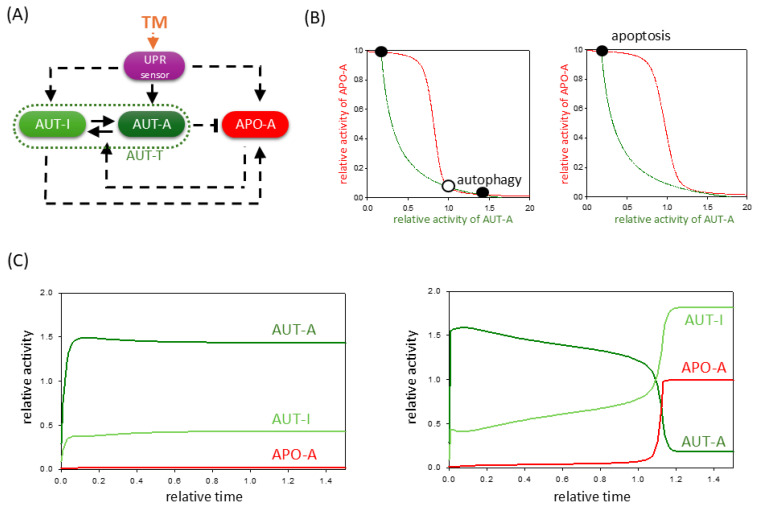
TM -induced ER stress acts indirectly on autophagy and apoptosis pathways via UPR. (**A**) The wiring diagram of the control network with respect to ER stress. TM indicates the effect of tunicamycin. The UPR sensor, autophagy inducer (AUT-A), and apoptosis inducer (APO-A) are grouped together in isolated purple, green, and red boxes, respectively. The active forms of the molecules are grouped in dark-colored boxes, while a light-colored box denotes the inactive form. Solid arrows represent biochemical reactions, and dashed lines show how the molecules can influence each other. Blocked end lines denote inhibition. (**B**) Phase plane diagrams are plotted upon (panel left) low (stress = 5) and (panel right) high (stress = 50) levels of TM stress. The balance curves of AUT-A (green) and APO-A (red) are plotted. Intersection of nullclines represents the stable (black dots) and unstable (unfilled circle) steady states. (**C**) The temporal dynamics of AUT-A, APO-A, and AUT-I are plotted upon low (panel left, stress = 5) and high (panel right, stress = 50) levels of ER stress.

Since TM specifically inhibits the proper protein folding by inhibiting protein glycosylation [33], our analysis hypothesizes that TM activates the upstream regulators of UPR (such as GRP78) [33]. Direct effects of TM on autophagy and apoptosis inducers have not yet been observed (see Table S2). Therefore, in our simple mathematical model, the effect of TM is incorporated by adding an extra positive term to the induction of the UPR sensor (Figure 2A), and then we verified the dynamic behavior of the system using phase plane analysis (Figure 2B). When we examine the signal response curves of both AUT-A and APO-A at two different TM levels, we suppose that at lower stress levels, autophagy is fully activated, but apoptotic cell death remains inactive (see the black dot labeled with “autophagy” in Figure 2B, panel left). In contrast, at a high level of TM, apoptosis becomes the only stable state of the system (see the black dot labeled with “apoptosis” in Figure 2B, panel right).

To further explore the dynamical behavior of the control network, computational simulations are presented even at low and high levels of TM treatments (Figure 2C). At a low concentration of TM, BCL-2 is quickly downregulated followed by a rapid, sigmoid activation of autophagy; meanwhile, APO-A remains inactive (Figure 2C, panel left). This result agrees with the experimental result when chondrocytes isolated from rat articular cartilage were treated with 0.5 μmol/L TM for 24 h [34]. It has shown that endoplasmic reticulum (ER) stress and the presence of autophagy (i.e., Beclin-1 and LC3-II) play important roles in the survival of chondrocytes even after 6 h of TM treatment. Similar results were observed when HepG2 cells were treated with low (1 μM) and high (100 μM) levels of TM for four hours [35].

In contrast, under high stress, we find that AUT-A has a transient upregulation while APO-A is still inactive, but after a while, APO-A is able to become active and in parallel rapidly inhibits autophagic survival. This behavior has been successfully demonstrated experimentally in TM treatment, when the level of LC3-II had a transient peak, while the pro-Caspase-3 level did not decrease, but after a while, Caspase-3 was able to become active and in parallel rapidly inhibits autophagic survival [35]. Our mathematical model also implies that there must be a concentration of TM in each cell type that acts as a threshold to ensure that apoptosis can be triggered or not.

Our theoretical study confirms that the dynamic behavior of TM is a consequence of the fact that TM acts only upstream of the inducers of both autophagy and apoptosis.

### 2.3. TG Induces UPR, but It Also Has a Direct Effect on Autophagy and Apoptosis Pathways

Thapsigargin is a well-known non-competitive inhibitor of the sarco/endoplasmic reticulum Ca^2+^ ATPase (SERCA), and this inhibition of the SERCA directly affects all three branches of the UPR pathway. Various experimental results have also demonstrated its ability to induce not only autophagy (i.e., via Beclin-1, Ca^2+^/calmodulin-dependent kinase kinase-beta, and AMPK) [36,37,38] but also apoptosis (i.e., mitochondrial-signaling-pathway-mediated apoptosis, death receptor 5, and Casp-8) genes [38,39].

**Figure 3:**
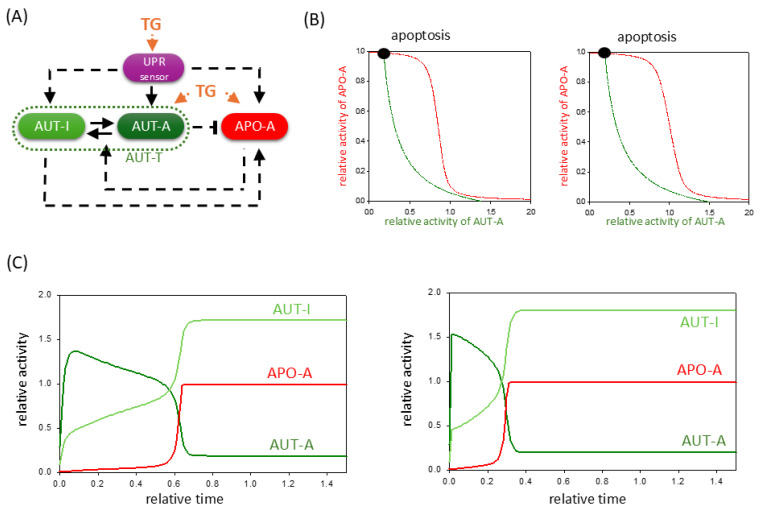
TG-induced ER stress acts indirectly on autophagy and apoptosis pathways via UPR. (**A**) The wiring diagram of the control network with respect to ER stress. TG indicates the effect of thapsigargin. The UPR sensor, autophagy inducer (AUT-A), and apoptosis inducer (APO-A) are grouped together in isolated purple, green, and red boxes, respectively. The active forms of the molecules are grouped in dark-colored boxes, while a light-colored box denotes the inactive form. Solid arrows represent biochemical reactions, and dashed lines show how the molecules can influence each other. Blocked end lines denote inhibition. (**B**) Phase plane diagrams are plotted upon (panel left) low (stress = 5, TG = 0.25) and (panel right) high (stress = 50, TG = 0.25) levels of TG stress. The balance curves of AUT-A (green) and APO-A (red) are plotted. Intersection of nullclines represents the stable (black dot) steady states. (**C**) The temporal dynamics of AUT-A, APO-A, and AUT-I are plotted upon low (panel left, stress = 5, TG = 0.25) and high (panel right, stress = 50, TG = 0.25) levels of ER stress.

Based on these experimental results, we assume that the TG has three attack points in our simple ER stress response model. Namely, we incorporated the effect of TG treatment in our model by directly activating AUT-A and APO-A in addition to inducing the UPR sensor (Figure 3A). First, we checked the complex effect of TG on the ER stress response with a phase plane diagram when treatments with both a lower and a higher TG concentration could be mimicked theoretically.

In both cases, although the AUT-A balance curve does not move drastically, the equilibrium curve of APO-A shifts more intensively to the right (Figure 3B). This results in the diminishment of the lower stable state even at a low level of TG, which would correspond to a stable autophagic response where apoptosis is inactive. Therefore, the system quickly moves to its only one stable state, where APO-A is high and AUTA-A is low, i.e., the cell induces apoptotic cell death. Time-course data also confirm that even with low concentrations of TG treatment, the cell cannot induce sustained autophagy; thus, it has no chance to save itself by self-digestion because AUT-A has only a transient activity peak (Figure 3C). However, despite TG’s positive effect on autophagy, the prompt activation of apoptotic pathways will inhibit the survival process through the double-negative feedback loop. These computer simulation results are consistent with previous experimental observations, where it was found that although TG can induce autophagy, it has a drastic effect on cell viability over time [38,39,40,41].

Our theoretical analysis confirms that a mild TG treatment is much more lethal than a mild TM treatment. Therefore, it is almost impossible to set a TG concentration level in the cell where only autophagic survival is activated because cell death is always triggered, even if with a delay.

### 2.4. The Negative Effect of ER Stressors Can Be Diminished by Hyperactivation of Autophagy Inducer

In the last couple of years, we have identified several natural compounds (such as resveratrol, EGCG (epigallocatechin gallate), and sulforaphane) that were able to induce autophagy-dependent survival upon ER stress via disrupting the balance between mTORC1 and AMPK pathways [41,42,43]. In our treatments, various human cells were pre-treated with one of the above-mentioned natural agents, and later ER stress was induced with either TG or TM [41,42,43]. We found that even with addition of an excessive level of either ER stressor, the transient autophagy peak persisted longer, and apoptosis could only be triggered much later in time. This effect of the autophagy inducer resulted in an increase in cell viability too, suggesting that prolonged autophagy might be essential in cell survival [41,42,43].

**Figure 4:**
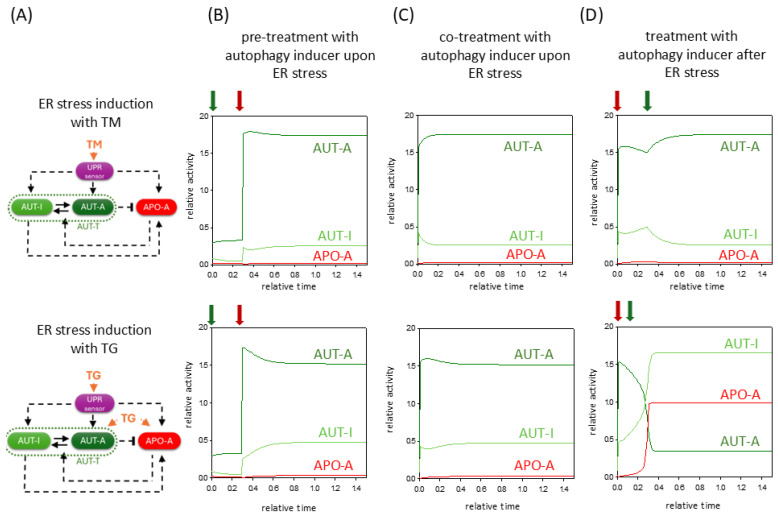
The negative effect of ER stressors can be diminished by hyperactivation of autophagy inducer. (**A**) The wiring diagram of the control network with respect to (upper panel) TM or (lower panel) TG treatment. The UPR sensor, autophagy inducer (AUT-A), and apoptosis inducer (APO-A) are grouped together in isolated purple, green, and red boxes, respectively. The active forms of the molecules are grouped in dark-colored boxes, while a light-colored box denotes the inactive form. Solid arrows represent biochemical reactions, and dashed lines show how the molecules can influence each other. Blocked end lines denote inhibition. The temporal dynamics of AUT-A, APO-A, and AUT-I are plotted in case of (**B**) pre-treatment or (**C**) co-treatment with autophagy inducer upon ER stress induction or (**D**) treatment with autophagy inducer after ER-stress-induced (upper panel) TM (kaua = 10, stress = 50) or (lower panel) TG (kaua = 10, stress = 50, TG = 0.25). Dark red arrows indicate the addition of the ER stressor; dark green arrows indicate the addition of the autophagy inducer.

Here, we investigate the dynamical characteristic of both pre-treatment and co-treatment with an autophagy inducer upon ER stress induced by either TG or TM (Figure 4B,C). Our time-series data suggest that both pre- and co-treatment with an autophagy inducer in the case of ER stress may allow AUT-A to keep the APO-A in an inactive state through the double-negative feedback loop between them. Although the ER stressor activates both autophagy and apoptosis via UPR, even during TG treatment directly via AUT-A and APO-A, with the help of the autophagy inducer, autophagy can predominate over apoptotic signaling, ensuring that cell viability is prolonged. Although TG has a more serious negative effect on cell survival compared to TM, an autophagy inducer chosen at the right concentration can rescue cells by self-digestion. Here, we would like to point out that the autophagy inducer in the case of either TM or TG treatment can increase the autophagy much more durably (Figure 4B,C).

We also used our theoretical analysis to investigate the effect of the autophagy inducer when we first induce an excessive level of ER stress and mimicked the addition of autophagy inducer with a certain time delay. While in TG treatment the cells induced apoptotic cell death without any rescue (Figure 4D, lower panel), in the TM treatment, we found a time window when, even with the addition of an autophagy inducer, the cells could rescue themselves by autophagy and not allow apoptosis to be triggered (Figure 4D, upper panel). However, if we tried to combine the TM treatment with the autophagy inducer at a later time point, the cells would have already switched irreversibly to a state of apoptotic cell death.

Our computational analysis suggests that pre-treatment or co-treatment with an autophagy inducer upon ER stress can significantly delay apoptotic cell death, thereby increasing cell viability. Our results also revealed that cells respond much worse to TG treatment than to TM treatment, with the effects of the former stressor being in many cases fatal for the cell.

### 2.5. Treatment with Different Concentrations of the Same Stressor Has a Cumulative Effect on the Response Mechanism

The ER stressor itself also induces autophagy. Even in the case of an excessive level of ER stress, the cell always tries to save itself by digesting the disrupted organelle first. The following question arises: whether pre-treatment of cells with such a concentration of ER stressor that only activates autophagy could postpone the activation of cell death pathways upon treatment with a fatal amount. Therefore, we particularly wanted to investigate whether the effects of the same ER stressor are additive or not.

**Figure 5:**
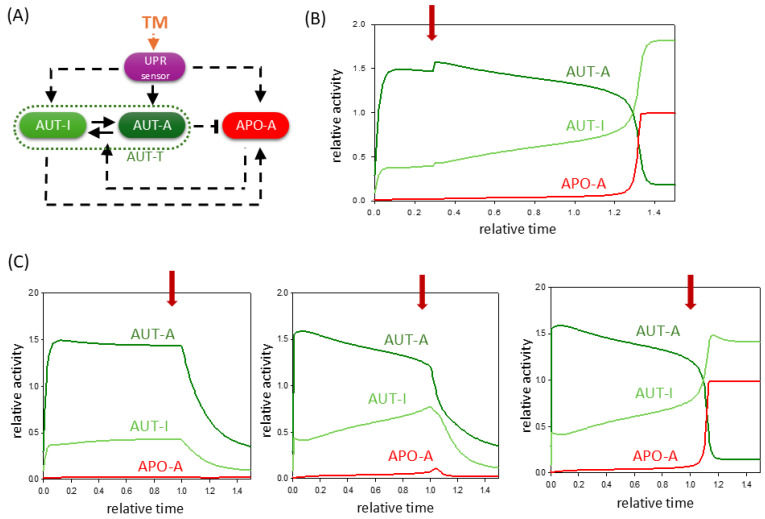
Treatment with different concentrations of the same stressor has a cumulative effect on the response mechanism. (**A**) The wiring diagram of the control network with respect to ER stress. TM indicates the effect of tunicamycin. The UPR sensor, autophagy inducer (AUT-A), and apoptosis inducer (APO-A) are grouped together in isolated purple, green, and red boxes, respectively. The active forms of the molecules are grouped in dark-colored boxes, while a light-colored box denotes the inactive form. Solid arrows represent biochemical reactions, and dashed lines show how the molecules can influence each other. Blocked end lines denote inhibition. (**B**) The temporal dynamics of AUT-A, APO-A, and AUT-I are plotted when low level of TM (stress = 5) was followed by high level of TM treatment (stress = 50). Dark red arrows indicate the addition of a high amount of ER stressor. (**C**) The temporal dynamics of AUT-A, APO-A, and AUT-I are plotted when (panel left) low level of TM (stress = 5) and (middle panel) and (panel right) high levels of TM (stress = 50) is reset to 0 (stress = 0), which the dark red arrow indicates.

To answer this question, we first mimicked a low-concentration TM treatment over time, which induced permanent autophagy in the cells (Figure 5B). After that, we increased the stress level to a high concentration and monitored its effect in time (see the dark red arrow in Figure 5B). Although we started from an autophagic state, in this case, AUT-A does not have a chance to prevail against APO-A. High TM also induces APO-A through the UPR, and the cells thus become switched in the process of cell death. These results confirm that treatment with the same ER stressor accumulates in the cell.

TG is so strongly bound to SERCA that it has an irreversible negative effect on the cell [44,45]; however, TM can be easily washed out from the cells [35,46]. Therefore, washing-out experiments can be used to model its effect on the stress response if ER stress is diminished for some reason (e.g., non-folded, damaged proteins get successfully cleared out). To mimic theoretically the washing out of TM from the cell, first low (stress = 5) or high (stress = 50) levels of TM are set, and then, after a certain time point, the value of stress is reset to zero (see dark red arrows in Figure 5C).

Our time course data highlight that by mimicking the wash out of a low concentration of TM, the cell can always return to its previous homeostasis (Figure 5C, panel left). In the case of ER stress induced by a high concentration of TM (Figure 5C, middle panel and panel right), the cell can only be saved until apoptosis is not induced. If ER stress is eliminated in the time window when autophagy is still active, the stressor can be reversibly washed out from the cells (Figure 5C, middle panel). If APO-A defeats AUT-A via the double-negative feedback loop, cell death processes get irreversibly activated and cells cannot be saved (Figure 5C, panel right). These results are in line with what we have already verified experimentally; namely, in the case of a high ER stressor with transient treatment, there is a temporal threshold value when the cells can still be saved. If the stressor is washed out later, apoptosis is irreversibly activated, while autophagy is switched off [35].

These results suggest to us that the effect of the ER stressor is cumulative, and its duration greatly influences the outcome of the stress response.

## 3. Discussion

Endoplasmic reticulum (ER) stress is involved in many common diseases, such as diabetes, neurodegenerative diseases, cancer, and many others [1,3,10,11]. Understanding the dynamical characteristics of the ER stress response pathways is crucial for developing successful medical treatment strategies. Recently, we developed a simple theoretical model of the cellular stress response mechanism [16]. Due to the positive and double-negative feedback loops of the control network, the cell could precisely choose between autophagy-dependent survival and apoptotic cell death [16]. Here, we redefine the simple network according to the crucial regulatory loops and connections of the ER stress response mechanism (Table S1). We claim that the same UPR sensor molecule induces both autophagy and apoptosis inducers upon ER stress, and the outcome of the decision-making process depends on the double-negative and positive feedback loops of the control network (Figure 1).

Our simple model is able to describe the dynamical characteristic of ER stress response both at tolerable and excessive levels of ER stress (Figure 1). By using various theoretical analysis (such as computational simulations, phase plane analysis, and signal response curves), we confirm here that ER-stress-induced UPR first always turns on autophagy-dependent self-digestion of damaged and unnecessary components to protect the cell from death. However, a non-tolerable level of ER stress results only in a transient peak of autophagy followed by apoptotic cell death (Figure 2). We suppose that in the case of an UPR-induced stress response, the cell always tries to rescue itself by autophagy and thus restore its internal homeostasis. However, upon a sustained level of stress, it is more beneficial for the whole organism to induce the death of the individual, but seriously damaged, cells.

These results are in complete agreement with previously published experimental data when different types of human cells were treated with the well-known ER stressors, such as tunicamycin (TM), thapsigargin (TG), and dithiothreitol (DTT) (Table S2). Our collection of results suggests that TM, DTT, and TG induce all the three branches of the UPR and thereby indirectly enhance both autophagy and apoptosis. For all the above-mentioned ER stressors, we found results demonstrating that under a low level of ER stress, cells attempt to rescue themselves by autophagy, whereas under an excessive level of ER stress, they turn on cell death pathways. However, our theoretical analysis also shows that there are fundamental differences in the effects of the three ER stressors on cells. While TM only activates autophagy and apoptosis via UPR, DTT has a direct influence on several apoptosis inducers (Table S2). Additionally, TG can directly induce both autophagy and apoptosis (Table S2). Our mathematical model suggests that these differences in their effects result in differences in the dynamic behavior of the ER stress response mechanism. Namely, cells are more sensitive to either TG or DTT treatment than the addition of TM. In the case of TG treatment, the double-negative feedback loop between autophagy and apoptosis inducers responds much more intensely to changes in stress levels, and although autophagy is activated quickly, there it has only a short transient upshift. Subsequently, the apoptosis inducer depresses the survival process and induces cell death (Figure 3). This is consistent with the fact that several experimental results contradict whether TG can induce autophagy-dependent survival in the cell for any length of time at all [47,48,49,50,51]. Our model confirms that cells are highly sensitive to TG treatments.

The exact role of autophagy in ER stress response was also investigated in the case of mimicking the addition of autophagy inducer before, together or after the treatment with TG or TM (Figure 4). In all cases, we claim that the window of autophagic survival is extended over time, allowing cells to survive more drastic ER stress. This is consistent with our previous experimental results when various natural compounds (such as resveratrol, EGCG, and sulforaphane) were used, which increased cell viability via autophagy induction and delayed the process of cell death in time [41,42,43]. Since the autophagy inducer can help increase cell viability even after ER stress has been induced, these natural compounds can be used for therapeutic purposes in diseases where we try to compensate the harmful effects of ER stress (e.g., neurodegenerative diseases and inflammatory bowel diseases) [52,53,54,55,56,57,58,59]. However, these ideas should be further explored in the near future.

Since autophagy might help cells to survive, we also wondered if turning on autophagy with the addition of a low level of the ER stressor could prevent the fatal effect of the treatment with an excessive amount of the same ER stressor. Our results suggest that the effect of the ER stressors is additive (Figure 5B). Since the stressor promotes both autophagy and apoptosis inducers, we suppose that we can only increase cell viability with a “pure” autophagy inducer. Another problem to model was the time dependency of the activation of survival versus cell death pathways or to investigate if cells can return to homeostasis after a certain time of ER stress activation. This can be tested experimentally by washing out the stressor molecule or theoretically by setting the stress levels to 0. Dynamic analysis shows that high ER stress turns on apoptosis irreversibly in time, and there is no return from a certain threshold point (Figure 5C). This result has been already experimentally verified using only DTT or TM as TG cannot be removed from the cells [35,45,46].

The consequence of DTT addition is even more drastic. Besides its promoting effect on UPR induction, its direct positive impact on the apoptosis inducer causes the cell survival window to virtually disappear, and the cells quickly go through apoptosis (Figure S1). Therefore, DTT can be a good candidate compound to improve the activation of cell death pathways [60]. For example, Bau et al. recently showed that DTT can be applied in cancer treatment precisely by inducing apoptosis [61]. They claimed that DTT could synergistically enhance the effects of treatment with As_2_O_3_ on eliminating oral cancer cells; meanwhile, DTT was non-toxic to the non-tumor cells. Therefore, DTT treatment seems to be promising for clinical practice in therapies of various types of cancer and worth further investigations.

Although TG also activates autophagy, its dynamic effect on the cell is more reminiscent of DTT than TM. This can be explained by TG’s direct impact on apoptosis induction due to the irreversible inhibition of SERCA, therefore, disrupting Ca^2+^ homeostasis. This strong cytotoxic potential placed it in the focus of anticancer therapy research, similar to DTT [40,62,63]. However, the serious disadvantage of TG is that it is not selective for tumor cells and shows significant toxicity towards healthy cells as well. Recently, various TG analogs or prodrugs have been introduced and tested that are effective in anticancer treatments in various tumor types but have no effect on normal cells [14,44,63]. For example, mipsagargin is a novel thapsigargin-based protease-activated pro-drug. In this compound, the thapsigargin analog molecule is linked to a peptide carrier that is a substrate of PMSA (prostate-specific membrane antigen). PMSA is a carboxypeptidase expressed only in prostate cancer cells or endothelial cells of solid tumors. This strategy allows for the deliberation of a TG-like molecule specifically in malignant tissues and induces their apoptosis. Compared to TG alone, mipsagargin can achieve higher concentrations of the active agent at the tumor site while avoiding systemic toxicity [14,64]. Several other TG analogs and derivatives showed promising preclinical results, and many of them have already entered clinical trials [62]. The differences and similarities between the three ER stressors presented here have been summarized in Table S3. Our goal was to give a simple guideline to compare the cellular response to the best-known ER-stress-provoking molecules, which might be useful in future experimental design.

Since our mathematical model includes many simplifications, we cannot ignore the need to carefully examine the limitations of our analysis. Although we have tried to process as much of the literature as possible (see Tables S1 and S2), some results may have been omitted by our mistake or new ones published since then. In our simplified model, we classified regulatory proteins of autophagy and apoptosis into three groups (AUT-A, AUT-I, and APO-A) and analyzed the dynamic connections between them. The possibility that there are other players in stress response control or other feedback loops describing their relation cannot be ruled out. The ER stressors presented here can also have additional points of attack in cellular systems, which may modify our results somewhat. We agree that more experimental support would better confirm the validity and accuracy of our analysis.

Setting clear aims is crucial for any medical treatments. Regarding cellular stress responses, the given condition can determine the need for cell death induction or the enforcement of survival pathways. ER-stress-inducing compounds can be selected accurately to support the desired therapeutic goal. According to our simplified model, when the therapeutic intention is to induce cell death (e.g., anticancer therapies), DTT or TG treatments are much more appropriate choices. If the aim is to force cells to increase their resistance through autophagy-dependent self-digestion (e.g., during various inflammatory diseases), the addition of TM in combination with an autophagy inducer can be an effective solution. We can conclude that our simple mathematical model exploring the dynamic behavior of the ER stress response brings us closer to understanding the mechanism of cellular outcomes, and we should take advantage of this knowledge in more precise therapeutic approaches in the future.

## 4. Materials and Methods

### Mathematical Modeling

Reaction rates in living systems are typically described using kinetic equations well known from chemical reaction kinetics; namely, a non-linear ordinary differential equation (ODE) can be established for the time-dependent rate variation of the concentration for each component. A generic differential equation describing the temporal changes of protein $X_{a}$ is composed of two parts: production and consumption terms.

$$dX_{a}/\mathrm{dt}=k_{s}+k_{act}*\left(X_{T}-X_{a}\right)-\left(k_{d}+k_{in}\right)*X_{a}$$

where

$X_{a}$—concentration of active *X*;

$X_{T}$—total concentration of *X*;

$k_{s}$— synthesis rate constant of *X*;

$k_{act}$—activation rate constant of $X_{a}$;

$k_{d}$—degradation rate constant of *X*;

$k_{in}$— inactivation rate constant of $X_{a}$.

Production of $X_{a}$ refers to the activation (e.g., post-translational modification) and/or de novo synthesis, while consumption represents the inactivation and/or degradation of protein *X*. Usually, synthesis, degradation, binding, and dissociation reactions are described by mass action kinetics, whereas protein activity can be described either by mass action or Michaelis–Menten kinetics [65,66]. For example, if the activity of protein is controlled by covalent modification involving multi-site phosphorylations, Michaelis–Menten kinetics provides a good approximation for the process [67,68]. The value of the parameters (rate constants and Michaelis constants) and the initial conditions have to be specified in order to solve the ODEs. The ensemble of equations constitutes a multi-parameter, nonlinear, first-order differential equation system. The non-linear nature of biological processes makes it difficult to find the solution of ODEs analytically; hence, the equations need to be solved numerically. The equations can be solved using different numerical integration methods that are implemented as solvers in many of the freely available computer software.

Solving a set of non-linear ODEs gives the time evolution of the relative protein concentration/activity (time courses). ODEs can be solved to obtain the input–output relationship called as signal response curves or as one parameter bifurcation diagram. Resolving this equation system, the results are compared to the behavior established in experimental way. If the results of the mathematical modeling are consistent with the experimental data, the given process could be visualized and interpreted [66]. In this work, the temporal profiles and signal response curves were computed numerical using XPP-AUT (freely available from https://sites.pitt.edu/~phase/bard/bardware/xpp/xpp.html (accessed on 6 November 2025)). All the simulations presented in the text are based on the following XPP codes: The rate constants (“k”) have the dimension of relative (time unit)^−1^, and the Michaelis constants (“J”) are dimensionless. The protein activities are given in arbitrary units (a.u). The starting parameter set was able to refer to physiological conditions. The parameter values were perturbed to capture all the possible qualitative behaviors that the given network can exhibit.

## Data Availability

The original contributions presented in this study are included in the article/Supplementary Materials. Further inquiries can be directed to the corresponding author.

## Supplementary Materials

The supporting information can be downloaded at https://www.mdpi.com/article/10.3390/ijms262210961/s1. References [6,9,12,17,18,19,20,21,22,23,24,25,26,27,28,29,30,31,32,33,34,36,37,38,39,40,44,60,69,70,71,72,73,74,75,76,77,78,79,80,81,82,83,84,85,86,87,88,89,90,91,92,93,94,95,96,97,98,99,100,101,102,103,104,105,106,107,108,109,110,111,112,113,114,115,116,117,118,119,120] are cited in the Supplementary Materials.

## Abbreviations

The following abbreviations are used in this manuscript:

ER	endoplasmic reticulum
UPR	unfolded protein response
AUT-A	autophagy inducer
APO-A	apoptosis inducer
TG	thapsigargin
TM	tunicamycin
DTT	dithiothreitol

## References

[1] da Silva D.C., Valentao P., Andrade P.B., Pereira D.M. (2020). Endoplasmic reticulum stress signaling in cancer and neurodegenerative disorders: Tools and strategies to understand its complexity. Pharmacol. Res..

[2] Schwarz D.S., Blower M.D. (2016). The endoplasmic reticulum: Structure, function and response to cellular signaling. Cell. Mol. Life Sci..

[3] Chen X., Shi C., He M., Xiong S., Xia X. (2023). Endoplasmic reticulum stress: Molecular mechanism and therapeutic targets. Signal Transduct. Target. Ther..

[4] Gorlach A., Klappa P., Kietzmann T. (2006). The endoplasmic reticulum: Folding, calcium homeostasis, signaling, and redox control. Antioxid. Redox Signal..

[5] Qi Z., Chen L. (2019). Endoplasmic Reticulum Stress and Autophagy. Adv. Exp. Med. Biol..

[6] Ogata M., Hino S., Saito A., Morikawa K., Kondo S., Kanemoto S., Murakami T., Taniguchi M., Tanii I., Yoshinaga K. (2006). Autophagy is activated for cell survival after endoplasmic reticulum stress. Mol. Cell. Biol..

[7] Tabas I., Ron D. (2011). Integrating the mechanisms of apoptosis induced by endoplasmic reticulum stress. Nat. Cell Biol..

[8] Spencer B.G., Finnie J.W. (2020). The Role of Endoplasmic Reticulum Stress in Cell Survival and Death. J. Comp. Pathol..

[9] Hoyer-Hansen M., Jaattela M. (2007). Connecting endoplasmic reticulum stress to autophagy by unfolded protein response and calcium. Cell Death Differ..

[10] Koksal A.R., Verne G.N., Zhou Q. (2021). Endoplasmic reticulum stress in biological processing and disease. J. Investig. Med..

[11] Kaneko M., Imaizumi K., Saito A., Kanemoto S., Asada R., Matsuhisa K., Ohtake Y. (2017). ER Stress and Disease: Toward Prevention and Treatment. Biol. Pharm. Bull..

[12] Wu J., Chen S., Liu H., Zhang Z., Ni Z., Chen J., Yang Z., Nie Y., Fan D. (2018). Tunicamycin specifically aggravates ER stress and overcomes chemoresistance in multidrug-resistant gastric cancer cells by inhibiting N-glycosylation. J. Exp. Clin. Cancer Res..

[13] Yoo J., Mashalidis E.H., Kuk A.C.Y., Yamamoto K., Kaeser B., Ichikawa S., Lee S.Y. (2018). GlcNAc-1-P-transferase-tunicamycin complex structure reveals basis for inhibition of N-glycosylation. Nat. Struct. Mol. Biol..

[14] Andersen T.B., Lopez C.Q., Manczak T., Martinez K., Simonsen H.T. (2015). Thapsigargin–from *Thapsia* L. to mipsagargin. Molecules.

[15] Oslowski C.M., Urano F. (2011). Measuring ER stress and the unfolded protein response using mammalian tissue culture system. Methods Enzymol..

[16] Kapuy O., Vinod P.K., Mandl J., Banhegyi G. (2013). A cellular stress-directed bistable switch controls the crosstalk between autophagy and apoptosis. Mol. Biosyst..

[17] Yamamoto K., Ichijo H., Korsmeyer S.J. (1999). BCL-2 is phosphorylated and inactivated by an ASK1/Jun N-terminal protein kinase pathway normally activated at G(2)/M. Mol. Cell. Biol..

[18] Siddiqui W.A., Ahad A., Ahsan H. (2015). The mystery of BCL2 family: Bcl-2 proteins and apoptosis: An update. Arch. Toxicol..

[19] Riedl S.J., Shi Y. (2004). Molecular mechanisms of caspase regulation during apoptosis. Nat. Rev. Mol. Cell Biol..

[20] Pattingre S., Tassa A., Qu X., Garuti R., Liang X.H., Mizushima N., Packer M., Schneider M.D., Levine B. (2005). Bcl-2 antiapoptotic proteins inhibit Beclin 1-dependent autophagy. Cell.

[21] Lindqvist L.M., Heinlein M., Huang D.C., Vaux D.L. (2014). Prosurvival Bcl-2 family members affect autophagy only indirectly, by inhibiting Bax and Bak. Proc. Natl. Acad. Sci. USA.

[22] Szegezdi E., Logue S.E., Gorman A.M., Samali A. (2006). Mediators of endoplasmic reticulum stress-induced apoptosis. EMBO Rep..

[23] Yang B., Liu Q., Bi Y. (2019). Autophagy and apoptosis are regulated by stress on Bcl2 by AMBRA1 in the endoplasmic reticulum and mitochondria. Theor. Biol. Med. Model..

[24] Strappazzon F., Vietri-Rudan M., Campello S., Nazio F., Florenzano F., Fimia G.M., Piacentini M., Levine B., Cecconi F. (2011). Mitochondrial BCL-2 inhibits AMBRA1-induced autophagy. EMBO J..

[25] Fimia G.M., Corazzari M., Antonioli M., Piacentini M. (2013). Ambra1 at the crossroad between autophagy and cell death. Oncogene.

[26] Ma P., Schwarten M., Schneider L., Boeske A., Henke N., Lisak D., Weber S., Mohrluder J., Stoldt M., Strodel B. (2013). Interaction of Bcl-2 with the autophagy-related GABAA receptor-associated protein (GABARAP): Biophysical characterization and functional implications. J. Biol. Chem..

[27] Siddiqui M.A., Mukherjee S., Manivannan P., Malathi K. (2015). RNase L Cleavage Products Promote Switch from Autophagy to Apoptosis by Caspase-Mediated Cleavage of Beclin-1. Int. J. Mol. Sci..

[28] Strappazzon F., Di Rita A., Cianfanelli V., D’Orazio M., Nazio F., Fimia G.M., Cecconi F. (2016). Prosurvival AMBRA1 turns into a proapoptotic BH3-like protein during mitochondrial apoptosis. Autophagy.

[29] Gordy C., He Y.W. (2012). The crosstalk between autophagy and apoptosis: Where does this lead?. Protein Cell.

[30] Zhu Y., Zhao L., Liu L., Gao P., Tian W., Wang X., Jin H., Xu H., Chen Q. (2010). Beclin 1 cleavage by caspase-3 inactivates autophagy and promotes apoptosis. Protein Cell.

[31] Kirsch D.G., Doseff A., Chau B.N., Lim D.S., de Souza-Pinto N.C., Hansford R., Kastan M.B., Lazebnik Y.A., Hardwick J.M. (1999). Caspase-3-dependent cleavage of Bcl-2 promotes release of cytochrome c. J. Biol. Chem..

[32] Zhu J., Yang Y., Wu J. (2007). Bcl-2 cleavages at two adjacent sites by different caspases promote cisplatin-induced apoptosis. Cell Res..

[33] Banerjee S., Ansari A.A., Upadhyay S.P., Mettman D.J., Hibdon J.R., Quadir M., Ghosh P., Kambhampati A., Banerjee S.K. (2024). Benefits and Pitfalls of a Glycosylation Inhibitor Tunicamycin in the Therapeutic Implication of Cancers. Cells.

[34] Wu H., Meng Z., Jiao Y., Ren Y., Yang X., Liu H., Wang R., Cui Y., Pan L., Cao Y. (2020). The endoplasmic reticulum stress induced by tunicamycin affects the viability and autophagy activity of chondrocytes. J. Clin. Lab. Anal..

[35] Holczer M., Marton M., Kurucz A., Banhegyi G., Kapuy O. (2015). A Comprehensive Systems Biological Study of Autophagy-Apoptosis Crosstalk during Endoplasmic Reticulum Stress. BioMed Res. Int..

[36] Hoyer-Hansen M., Bastholm L., Szyniarowski P., Campanella M., Szabadkai G., Farkas T., Bianchi K., Fehrenbacher N., Elling F., Rizzuto R. (2007). Control of macroautophagy by calcium, calmodulin-dependent kinase kinase-beta, and Bcl-2. Mol. Cell.

[37] Grotemeier A., Alers S., Pfisterer S.G., Paasch F., Daubrawa M., Dieterle A., Viollet B., Wesselborg S., Proikas-Cezanne T., Stork B. (2010). AMPK-independent induction of autophagy by cytosolic Ca^2+^ increase. Cell Signal..

[38] Wang C., Li T., Tang S., Zhao D., Zhang C., Zhang S., Deng S., Zhou Y., Xiao X. (2016). Thapsigargin induces apoptosis when autophagy is inhibited in HepG2 cells and both processes are regulated by ROS-dependent pathway. Environ. Toxicol. Pharmacol..

[39] Lindner P., Christensen S.B., Nissen P., Moller J.V., Engedal N. (2020). Cell death induced by the ER stressor thapsigargin involves death receptor 5, a non-autophagic function of MAP1LC3B, and distinct contributions from unfolded protein response components. Cell Commun. Signal..

[40] Jaskulska A., Janecka A.E., Gach-Janczak K. (2020). Thapsigargin-From Traditional Medicine to Anticancer Drug. Int. J. Mol. Sci..

[41] Holczer M., Banhegyi G., Kapuy O. (2016). GADD34 Keeps the mTOR Pathway Inactivated in Endoplasmic Reticulum Stress Related Autophagy. PLoS ONE.

[42] Holczer M., Besze B., Lehel A., Kapuy O. (2024). The Dual Role of Sulforaphane-Induced Cellular Stress-A Systems Biological Study. Int. J. Mol. Sci..

[43] Holczer M., Besze B., Zambo V., Csala M., Banhegyi G., Kapuy O. (2018). Epigallocatechin-3-Gallate (EGCG) Promotes Autophagy-Dependent Survival via Influencing the Balance of mTOR-AMPK Pathways upon Endoplasmic Reticulum Stress. Oxid. Med. Cell. Longev..

[44] Sehgal P., Szalai P., Olesen C., Praetorius H.A., Nissen P., Christensen S.B., Engedal N., Moller J.V. (2017). Inhibition of the sarco/endoplasmic reticulum (ER) Ca^2+^-ATPase by thapsigargin analogs induces cell death via ER Ca^2+^ depletion and the unfolded protein response. J. Biol. Chem..

[45] Lytton J., Westlin M., Hanley M.R. (1991). Thapsigargin inhibits the sarcoplasmic or endoplasmic reticulum Ca-ATPase family of calcium pumps. J. Biol. Chem..

[46] Grabel L.B., Martin G.R. (1983). Tunicamycin reversibly inhibits the terminal differentiation of teratocarcinoma stem cells to endoderm. Dev. Biol..

[47] Engedal N., Torgersen M.L., Guldvik I.J., Barfeld S.J., Bakula D., Saetre F., Hagen L.K., Patterson J.B., Proikas-Cezanne T., Seglen P.O. (2013). Modulation of intracellular calcium homeostasis blocks autophagosome formation. Autophagy.

[48] Ganley I.G., Wong P.M., Gammoh N., Jiang X. (2011). Distinct autophagosomal-lysosomal fusion mechanism revealed by thapsigargin-induced autophagy arrest. Mol. Cell.

[49] Dubois C., Kondratskyi A., Bidaux G., Noyer L., Vancauwenberghe E., Farfariello V., Toillon R.A., Roudbaraki M., Tierny D., Bonnal J.L. (2020). Co-targeting Mitochondrial Ca^2+^ Homeostasis and Autophagy Enhances Cancer Cells’ Chemosensitivity. iScience.

[50] Williams A., Sarkar S., Cuddon P., Ttofi E.K., Saiki S., Siddiqi F.H., Jahreiss L., Fleming A., Pask D., Goldsmith P. (2008). Novel targets for Huntington’s disease in an mTOR-independent autophagy pathway. Nat. Chem. Biol..

[51] Gordon P.B., Holen I., Fosse M., Rotnes J.S., Seglen P.O. (1993). Dependence of hepatocytic autophagy on intracellularly sequestered calcium. J. Biol. Chem..

[52] Mbara K.C., Fotsing M.C.D., Ndinteh D.T., Mbeb C.N., Nwagwu C.S., Khan R., Mokhetho K.C., Baijnath H., Nlooto M., Mokhele S. (2024). Endoplasmic reticulum stress in pancreatic beta-cell dysfunction: The potential therapeutic role of dietary flavonoids. Curr. Res. Pharmacol. Drug Discov..

[53] Al Azzani M., Nizami Z.N., Magramane R., Sekkal M.N., Eid A.H., Al Dhaheri Y., Iratni R. (2024). Phytochemical-mediated modulation of autophagy and endoplasmic reticulum stress as a cancer therapeutic approach. Phytother. Res..

[54] Hajimohammadi S., Rameshrad M., Karimi G. (2024). Exploring the therapeutic effects of sulforaphane: An in-depth review on endoplasmic reticulum stress modulation across different disease contexts. Inflammopharmacology.

[55] Mansour S.Z., Moustafa E.M., Moawed F.S.M. (2022). Modulation of endoplasmic reticulum stress via sulforaphane-mediated AMPK upregulation against nonalcoholic fatty liver disease in rats. Cell Stress Chaperones.

[56] Dana A.H., Alejandro S.P. (2022). Role of sulforaphane in endoplasmic reticulum homeostasis through regulation of the antioxidant response. Life Sci..

[57] Ding S., Jiang J., Zhang G., Bu Y., Zhang G., Zhao X. (2017). Resveratrol and caloric restriction prevent hepatic steatosis by regulating SIRT1-autophagy pathway and alleviating endoplasmic reticulum stress in high-fat diet-fed rats. PLoS ONE.

[58] Gowd V., Kang Q., Wang Q., Wang Q., Chen F., Cheng K.W. (2020). Resveratrol: Evidence for Its Nephroprotective Effect in Diabetic Nephropathy. Adv. Nutr..

[59] Yuan D., Liu X.M., Fang Z., Du L.L., Chang J., Lin S.H. (2018). Protective effect of resveratrol on kidney in rats with diabetic nephropathy and its effect on endoplasmic reticulum stress. Eur. Rev. Med. Pharmacol. Sci..

[60] Xiang X.Y., Yang X.C., Su J., Kang J.S., Wu Y., Xue Y.N., Dong Y.T., Sun L.K. (2016). Inhibition of autophagic flux by ROS promotes apoptosis during DTT-induced ER/oxidative stress in HeLa cells. Oncol. Rep..

[61] Tsai C.W., Chang N.W., Tsai R.Y., Wang R.F., Hsu C.M., Lin S.S., Wu C.N., Sun S.S., Tsai M.H., Bau D.T. (2010). Synergistic cytotoxic effects of arsenic trioxide plus dithiothreitol on mice oral cancer cells. Anticancer Res..

[62] Khurram I., Khan M.U., Ibrahim S., Ghani M.U., Amin I., Falzone L., Herrera-Bravo J., Setzer W.N., Sharifi-Rad J., Calina D. (2024). Thapsigargin and its prodrug derivatives: Exploring novel approaches for targeted cancer therapy through calcium signaling disruption. Med. Oncol..

[63] Suresh A., Bagchi D., Kaliappan K.P. (2024). Thapsigargin: A promising natural product with diverse medicinal potential - a review of synthetic approaches and total syntheses. Org. Biomol. Chem..

[64] Christensen S.B., Simonsen H.T., Engedal N., Nissen P., Moller J.V., Denmeade S.R., Isaacs J.T. (2021). From Plant to Patient: Thapsigargin, a Tool for Understanding Natural Product Chemistry, Total Syntheses, Biosynthesis, Taxonomy, ATPases, Cell Death, and Drug Development. Prog. Chem. Org. Nat. Prod..

[65] Tyson J.J., Chen K.C., Novak B. (2003). Sniffers, buzzers, toggles and blinkers: Dynamics of regulatory and signaling pathways in the cell. Curr. Opin. Cell Biol..

[66] Tyson J.J., Csikasz-Nagy A., Novak B. (2002). The dynamics of cell cycle regulation. BioEssays.

[67] Ferrell J.E. (1996). Tripping the switch fantastic: How a protein kinase cascade can convert graded inputs into switch-like outputs. Trends Biochem. Sci..

[68] Kapuy O., Barik D., Sananes M.R., Tyson J.J., Novak B. (2009). Bistability by multiple phosphorylation of regulatory proteins. Prog. Biophys. Mol. Biol..

[69] Bassik M.C., Scorrano L., Oakes S.A., Pozzan T., Korsmeyer S.J. (2004). Phosphorylation of BCL-2 regulates ER Ca^2+^ homeostasis and apoptosis. EMBO J..

[70] Bhatt K., Feng L., Pabla N., Liu K., Smith S., Dong Z. (2008). Effects of targeted Bcl-2 expression in mitochondria or endoplasmic reticulum on renal tubular cell apoptosis. Am. J. Physiol. Renal Physiol..

[71] Brahmbhatt H., Oppermann S., Osterlund E.J., Leber B., Andrews D.W. (2015). Molecular Pathways: Leveraging the BCL-2 Interactome to Kill Cancer Cells–Mitochondrial Outer Membrane Permeabilization and Beyond. Clin. Cancer Res..

[72] Chandrika B.B., Yang C., Ou Y., Feng X., Muhoza D., Holmes A.F., Theus S., Deshmukh S., Haun R.S., Kaushal G.P. (2015). Endoplasmic Reticulum Stress-Induced Autophagy Provides Cytoprotection from Chemical Hypoxia and Oxidant Injury and Ameliorates Renal Ischemia-Reperfusion Injury. PLoS ONE.

[73] Chang N.C., Nguyen M., Germain M., Shore G.C. (2010). Antagonism of Beclin 1-dependent autophagy by BCL-2 at the endoplasmic reticulum requires NAF-1. EMBO J..

[74] Ciechomska I.A., Goemans G.C., Skepper J.N., Tolkovsky A.M. (2009). Bcl-2 complexed with Beclin-1 maintains full anti-apoptotic function. Oncogene.

[75] Djavaheri-Mergny M., Maiuri M. C., Kroemer G. (2010). Cross talk between apoptosis and autophagy by caspase-mediated cleavage of Beclin 1. Oncogene.

[76] G G., Singh J. (2022). Dithiothreitol causes toxicity in C. elegans by modulating the methionine-homocysteine cycle. eLife.

[77] Gross A., Katz S.G. (2017). Non-apoptotic functions of BCL-2 family proteins. Cell Death Differ..

[78] Guha P., Kaptan E., Gade P., Kalvakolanu D.V., Ahmed H. (2017). Tunicamycin induced endoplasmic reticulum stress promotes apoptosis of prostate cancer cells by activating mTORC1. Oncotarget.

[79] Hacki J., Egger L., Monney L., Conus S., Rosse T., Fellay I., Borner C. (2000). Apoptotic crosstalk between the endoplasmic reticulum and mitochondria controlled by Bcl-2. Oncogene.

[80] Heath-Engel H.M., Chang N.C., Shore G.C. (2008). The endoplasmic reticulum in apoptosis and autophagy: Role of the BCL-2 protein family. Oncogene.

[81] Held K.D., Melder D.C. (1987). Toxicity of the sulfhydryl-containing radioprotector dithiothreitol. Radiat Res..

[82] Held K.D., Sylvester F.C., Hopcia K.L., Biaglow J.E. (1996). Role of Fenton chemistry in thiol-induced toxicity and apoptosis. Radiat. Res..

[83] Hou W., Han J., Lu C., Goldstein L.A., Rabinowich H. (2010). Autophagic degradation of active caspase-8: A crosstalk mechanism between autophagy and apoptosis. Autophagy.

[84] Huang X., Qi Q., Hua X., Li X., Zhang W., Sun H., Li S., Wang X., Li B. (2014). Beclin 1, an autophagy-related gene, augments apoptosis in U87 glioblastoma cells. Oncol. Rep..

[85] Kang R., Zeh H.J., Lotze M.T., Tang D. (2011). The Beclin 1 network regulates autophagy and apoptosis. Cell Death Differ..

[86] Kania E., Pajak B., Orzechowski A. (2015). Calcium homeostasis and ER stress in control of autophagy in cancer cells. BioMed Res. Int..

[87] Keestra-Gounder A.M., Byndloss M.X., Seyffert N., Young B.M., Chavez-Arroyo A., Tsai A.Y., Cevallos S.A., Winter M.G., Pham O.H., Tiffany C.R. (2016). NOD1 and NOD2 signalling links ER stress with inflammation. Nature.

[88] Lam M., Lawrence D.A., Ashkenazi A., Walter P. (2018). Confirming a critical role for death receptor 5 and caspase-8 in apoptosis induction by endoplasmic reticulum stress. Cell Death Differ..

[89] Lee J.H., Rho S.B., Chun T. (2005). GABAA receptor-associated protein (GABARAP) induces apoptosis by interacting with DEAD (Asp-Glu-Ala-Asp/His) box polypeptide 47 (DDX 47). Biotechnol. Lett..

[90] Lei K., Davis R.J. (2003). JNK phosphorylation of Bim-related members of the Bcl2 family induces Bax-dependent apoptosis. Proc. Natl. Acad. Sci. USA.

[91] Li X., Su J., Xia M., Li H., Xu Y., Ma C., Ma L., Kang J., Yu H., Zhang Z. (2016). Caspase-mediated cleavage of Beclin1 inhibits autophagy and promotes apoptosis induced by S1 in human ovarian cancer SKOV3 cells. Apoptosis.

[92] Lu M., Lawrence D.A., Marsters S., Acosta-Alvear D., Kimmig P., Mendez A.S., Paton A.W., Paton J.C., Walter P., Ashkenazi A. (2014). Opposing unfolded-protein-response signals converge on death receptor 5 to control apoptosis. Science.

[93] Luhr M., Torgersen M.L., Szalai P., Hashim A., Brech A., Staerk J., Engedal N. (2019). The kinase PERK and the transcription factor ATF4 play distinct and essential roles in autophagy resulting from tunicamycin-induced ER stress. J. Biol. Chem..

[94] Luo B., Lee A.S. (2013). The critical roles of endoplasmic reticulum chaperones and unfolded protein response in tumorigenesis and anticancer therapies. Oncogene.

[95] Luo S., Rubinsztein D.C. (2010). Apoptosis blocks Beclin 1-dependent autophagosome synthesis: An effect rescued by Bcl-xL. Cell Death Differ..

[96] Ma Z., Fan C., Yang Y., Di S., Hu W., Li T., Zhu Y., Han J., Xin Z., Wu G. (2016). Thapsigargin sensitizes human esophageal cancer to TRAIL-induced apoptosis via AMPK activation. Sci. Rep..

[97] Maiuri M.C., Le Toumelin G., Criollo A., Rain J.C., Gautier F., Juin P., Tasdemir E., Pierron G., Troulinaki K., Tavernarakis N. (2007). Functional and physical interaction between Bcl-X(L) and a BH3-like domain in Beclin-1. EMBO J..

[98] Marquez R.T., Xu L. (2012). Bcl-2:Beclin 1 complex: Multiple, mechanisms regulating autophagy/apoptosis toggle switch. Am. J. Cancer Res..

[99] McCullough K.D., Martindale J.L., Klotz L.O., Aw T.Y., Holbrook N.J. (2001). Gadd153 sensitizes cells to endoplasmic reticulum stress by down-regulating Bcl2 and perturbing the cellular redox state. Mol. Cell Biol..

[100] Munoz-Pinedo C., Lopez-Rivas A. (2018). A role for caspase-8 and TRAIL-R2/DR5 in ER-stress-induced apoptosis. Cell Death Differ..

[101] Oakes S.A., Lin S.S., Bassik M.C. (2006). The control of endoplasmic reticulum-initiated apoptosis by the BCL-2 family of proteins. Curr. Mol. Med..

[102] Pihan P., Carreras-Sureda A., Hetz C. (2017). BCL-2 family: Integrating stress responses at the ER to control cell demise. Cell Death Differ..

[103] Puthalakath H., O’Reilly L.A., Gunn P., Lee L., Kelly P.N., Huntington N.D., Hughes P.D., Michalak E.M., McKimm-Breschkin J., Motoyama N. (2007). ER stress triggers apoptosis by activating BH3-only protein Bim. Cell.

[104] Rashid H.O., Yadav R.K., Kim H.R., Chae H.J. (2015). ER stress: Autophagy induction, inhibition and selection. Autophagy.

[105] Ravi, Kumar A., Bhattacharyya S., Singh J. (2023). Thiol reductive stress activates the hypoxia response pathway. EMBO J..

[106] Rodriguez D., Rojas-Rivera D., Hetz C. (2011). Integrating stress signals at the endoplasmic reticulum: The BCL-2 protein family rheostat. Biochim. Biophys. Acta Mol. Cell Res..

[107] Rong Y.P., Bultynck G., Aromolaran A.S., Zhong F., Parys J.B., De Smedt H., Mignery G.A., Roderick H.L., Bootman M.D., Distelhorst C.W. (2009). The BH4 domain of Bcl-2 inhibits ER calcium release and apoptosis by binding the regulatory and coupling domain of the IP3 receptor. Proc. Natl. Acad. Sci. USA.

[108] Sakaki K., Wu J., Kaufman R.J. (2008). Protein kinase Ctheta is required for autophagy in response to stress in the endoplasmic reticulum. J. Biol. Chem..

[109] Sano R., Reed J.C. (2013). ER stress-induced cell death mechanisms. Biochim. Biophys. Acta Mol. Cell Res..

[110] Sramek J., Nemcova-Furstova V., Kovar J. (2021). Molecular Mechanisms of Apoptosis Induction and Its Regulation by Fatty Acids in Pancreatic beta-Cells. Int. J. Mol. Sci..

[111] Szegezdi E., Macdonald D.C., Ni Chonghaile T., Gupta S., Samali A. (2009). Bcl-2 family on guard at the ER. Am. J. Physiol. Cell Physiol..

[112] Tartier L., McCarey Y.L., Biaglow J.E., Kochevar I.E., Held K.D. (2000). Apoptosis induced by dithiothreitol in HL-60 cells shows early activation of caspase 3 and is independent of mitochondria. Cell Death Differ..

[113] Vicencio J.M., Ortiz C., Criollo A., Jones A.W., Kepp O., Galluzzi L., Joza N., Vitale I., Morselli E., Tailler M. (2009). The inositol 1,4,5-trisphosphate receptor regulates autophagy through its interaction with Beclin 1. Cell Death Differ..

[114] Wang Y., Zhang L., He Z., Deng J., Zhang Z., Liu L., Ye W., Liu S. (2020). Tunicamycin induces ER stress and inhibits tumorigenesis of head and neck cancer cells by inhibiting N-glycosylation. Am. J. Transl. Res..

[115] Wei Y., Pattingre S., Sinha S., Bassik M., Levine B. (2008). JNK1-mediated phosphorylation of Bcl-2 regulates starvation-induced autophagy. Mol. Cell.

[116] Wirawan E., Vande Walle L., Kersse K., Cornelis S., Claerhout S., Vanoverberghe I., Roelandt R., De Rycke R., Verspurten J., Declercq W. (2010). Caspase-mediated cleavage of Beclin-1 inactivates Beclin-1-induced autophagy and enhances apoptosis by promoting the release of proapoptotic factors from mitochondria. Cell Death Dis..

[117] Yamaguchi H., Wang H.G. (2004). CHOP is involved in endoplasmic reticulum stress-induced apoptosis by enhancing DR5 expression in human carcinoma cells. J. Biol. Chem..

[118] Yang X., Srivastava R., Howell S.H., Bassham D.C. (2016). Activation of autophagy by unfolded proteins during endoplasmic reticulum stress. Plant J..

[119] Yorimitsu T., Nair U., Yang Z., Klionsky D.J. (2006). Endoplasmic reticulum stress triggers autophagy. J. Biol. Chem..

[120] Zong W.X., Li C., Hatzivassiliou G., Lindsten T., Yu Q.C., Yuan J., Thompson C.B. (2003). Bax and Bak can localize to the endoplasmic reticulum to initiate apoptosis. J. Cell Biol..

