# Supplementary material for: Fine-Tuning of the Endoplasmic Reticulum Stress Response Mechanism Plays a Key Role in Cellular Survival—A Mathematical Study
Source: Int J Mol Sci. 2025 Nov 12;26(22):10961. doi: 10.3390/ijms262210961 (PMC12652979; doi:10.3390/ijms262210961)
Supplement: Supplementary file 1 [file ijms-26-10961-s001.zip › main.pdf]

Supplementary Information
"The fine-tuning of endoplasmic reticulum stress response mechanism plays a key role in cellular survival – a mathematical study"

Marianna Holczer[1], Margita Márton [1], Ibolya Stiller[1], Beáta Lizák[1], Gábor Bánhegyi[1] and Orsolya Kapuy [1]

[1] Semmelweis University, Department of Molecular Biology, Institute of Biochemistry and Molecular Biology, Budapest, Hungary

1 Mathematical codes for computational simulations

1.1 The code for time course simulations

differential equations
Bcl2 represents the active form of BCL2
Bcl2' = kbcs - (kbcd + kbcd'*stress + kbcd"*APOA)*Bcl2

Bax represents the active form of BAX
Bax' = kbaa*(Baxt-Bax) - (kbai + kbai'*Bcl2 + kbai"*APOA)*Bax

mTOR represents the active form of mTORC1
mTOR' = (kamtor + kamtor'*ERS)*(mTORT-mTOR) - (kimtor + beta*SFN + kimtor'*Auta + kimtor"*Ind)* mTOR

APOA represents the active form of apoptosis inducer (APO-A)
APOA' = (kapa + kapa'*AUTI + kapa"*Bax + kapa"'*TG)*(APOT-APOA)/(Jap + APOT-APOA) - (kapi + kapi'*AUTA*APOA/(Jap + APOA

AUTA represents the active, not complex bound form of autophagy inducer (AUT-A)
Auta' = - kasau*(Bcl2 - AUTAC - AUTIC)*AUTA + (kdsau + kbau + kbau'*stress + kbau"*APOA)*AUTAC + (kaua + kaua'*TG)*AUTI - (kaui + kaui'*APOA + kaui"*TG)*AUTA

AUTI represents the inactive, not complex bound form of autophagy inducer (AUT-I)
AUTI' = - kasau*(Bcl2 - AUTAC - AUTIC)*AUTI + (kdsau + kbau + kbau'*stress + kbau"*APOA)*AUTAC - (kaua + kaua'*TG)*AUTI + (kaui + kaui'*APOA + kaui"*TG)*AUTA

AUTAC represents the active Beclin1 bound by BCL2
AUTAC' = kasau*(Bcl2 - AUTAC - AUTIC)*AUTA - (kdsau + kbau + kbau'*stress

+ kbau”*APOA)*AUTAC + kaua + kaua’*TG)*AUTIC - (kaui + kaui’*Casp + kaui”*TG)*AUTAC

# Becic represents the inactive form of Beclin1 bound by BCL2
AUTIC = AUTT - AUTA - AUTI - AUTAC
aux AUTIC = AUTIC

parameters
low TM treatment: stress=5
high TM treatment: stress=50
low TG treatment: stress=5, TG=0.25
high TG treatment: stress=50, TG=0.25
low DTT treatment: stress=5, kapa=5
high DTT treatment: stress=50, kapa=5
autophagy inducer treatment: kaua=10
p stress=0, TG=0
p kbcs=10, kbcd=5, kbcd’=10, kbcd”=15
p kbaa=20, kbai=0.1, kbai’=20, kbai”=5, BaxT=1
p p kaua=5, kaua’=2, kaui=0.5, kaui’=50, kaui”=5, AUTT=1, kdsau=1, kasau=100
p Jkapa=0.01, kapa’=10, kapa”=150, kapa”’=15, kapi=50, kapi’=100, APOT=1, Jap=0.01

done

1.2 The code for simulating signal-response curves

1.2.1 The signal-response curve of AUT-A with respect to the increasing stress level

differential equations
AUTA represents the active form of autophagy inducer (AUT-A)
AUTA’ = AUTA - AUTAA

stress represent the increasing stress level
stress’ = 0

# steady state functions
# Bcl2 represents the active form of BCL2
Bcl2 = kbcs / (kbcd + kbcd’*stress + kbcd”*APOA)

# Bax represents the active form of BAX
Bax = kbaa*BaxT / (kbaa + kbai + kbai’*Bcl2 + kbai”*APOA)

# AUTAT represents that form of AUTA which is not complex bound
AUTAT = (kaua + kaua’*TG)*AUTT / (kaua + kaua’*TG + kaui+kaui’*APOA+kaui”*TG)

# algebraic calculation of various forms of autophagy inducer
BB = AUTT + Bcl2 + kdiss/kass
C = 2*AUTT*Bcl2/(BB + sqrt(BB^2 - 4*AUTT*Bcl2))

```
AUTAA= AUTAT*(AUTT-C)/AUTT
AUTI = (AUTT-AUTAT)*(AUTT-C)/AUTT

# APOA represents the active form of apoptosis inducer (APO-A)
APOA= APOT*GK(kapa + kapa’*AUTI + kapa”*Bax + kapa”’*TG,kapi + kapi”*AUTA,Jap,Jap)

# ’Goldbeter-Koshand’ function (GK)
GB(arg1,arg2,arg3,arg4) = arg2-arg1+arg2*arg3+arg1*arg4
GK(arg1,arg2,arg3,arg4) = 2*arg1*arg4/(GB(arg1,arg2,arg3,arg4)+
sqrt(GB(arg1,arg2,arg3,arg4)ˆ2-4*(arg2-arg1)*arg1*arg4))

# parameters
p TG=0
p kbcs=10, kbcd=5, kbcd’=10, kbcd”=15
p kbaa=20, kbai=0.1, kbai’=20, kbai”=5, BaxT=1
p kaua=5, kaua’=2, kaui=0.5, kaui’=50, kaui”=5, AUTT=1, kdiss=1, kass=100
p kapa=0.01, kapa’=10, kapa”=150, kapa”’=15, kapi=50, kapi”=100, APOT=1,
Jap=0.01

done
```

1.2.2 The signal-response curve of APO-A with respect to the increasing stress level

```
# differential equations
# APOA represents the active form of apoptosis inducer (APO-A)
APOA’ = (kapa + kapa’* AUTI + kapa”*Bax + kapa”’*TG)*(APOT-APOA)/(Jap
+ APOT-APOA) - (kapi + kapi”*AUTA)*APOA/(Jap + APOA)

# stress represent the increasing stress level
stress’ = 0

# steady state function
# Bcl2 represents the active form of BCL2
Bcl2 = kbcs / (kbcd + kbcd’*stress + kbcd”*APOA)

# Bax represents the active form of BAX
Bax = kbaa*BaxT / (kbaa + kbai + kbai’*Bcl2 + kbai”*APOA)

# AUTAT represents that form of AUTA which is not complex bound
AUTAT = (kaua + kaua’*TG)*AUTT / (kaua + kaua’*TG + kaui+kaui’*APOA+kaui”*TG)

# algebraic calculation of various forms of autophagy inducer
BB = AUTT + Bcl2 + kdiss/kass
C = 2*AUTT*Bcl2/(BB + sqrt(BBˆ2 - 4*AUTT*Bcl2))
AUTAA= AUTAT*(AUTT-C)/AUTT
AUTI = (AUTT-AUTAT)*(AUTT-C)/AUTT
```

```
# 'Goldbeter-Koshand' function (GK)
GB(arg1,arg2,arg3,arg4) = arg2-arg1+arg2*arg3+arg1*arg4
GK(arg1,arg2,arg3,arg4) = 2*arg1*arg4/(GB(arg1,arg2,arg3,arg4)+
sqrt(GB(arg1,arg2,arg3,arg4)^2-4*(arg2-arg1)*arg1*arg4))

# parameters
p TG=0
p kbcs=10, kbcd=5, kbcd'=10, kbcd"=15
p kbaa=20, kbai=0.1, kbai'=20, kbai"=5, BaxT=1
p kaua=5, kaua'=2, kaui=0.5, kaui'=50, kaui"=5, AUTT=2, kdiss=1, kass=100
p kapa=0.01, kapa'=10, kapa"=150, kapa'"=15, kapi=50, kapi"=100, APOT=1,
Jap=0.01

 done
```

1.3 The code for simulating phase plane diagrams

1.3.1 The signal-response curve of AUTA-A with respect to APO-A

```
# differential equations
# AUTA represents the active form of autophagy inducer (AUT-A)
AUTA' = AUTA - AUTAA

# APOA represents the active form of apoptosis inducer (APO-A)
APOA' = (kapa + kapa'* AUTI + kapa"*Bax + kapa'"*TG)*(APOT-APOA)/(Jap
+ APOT-APOA) - (kapi + kapi"*AUTA)*APOA/(Jap + APOA)

# steady state function
# Bcl2 represents the active form of BCL2
Bcl2 = kbcs / (kbcd + kbcd'*stress + kbcd"*APOA)

# Bax represents the active form of BAX
Bax = kbaa*BaxT / (kbaa + kbai + kbai'*Bcl2 + kbai"*APOA)

# AUTAT represents that form of AUTA which is not complex bound
AUTAT = (kaua + kaua'*TG)*AUTT / (kaua + kaua'*TG + kaui+kaui'*APOA+kaui"*TG)

# algebraic calculation of various forms of autophagy inducer
BB = AUTT + Bcl2 + kdiss/kass
C = 2*AUTT*Bcl2/(BB + sqrt(BB^2 - 4*AUTT*Bcl2))
AUTAA= AUTAT*(AUTT-C)/AUTT
AUTI = (AUTT-AUTAT)*(AUTT-C)/AUTT

# 'Goldbeter-Koshand' function (GK)
GB(arg1,arg2,arg3,arg4) = arg2-arg1+arg2*arg3+arg1*arg4
GK(arg1,arg2,arg3,arg4) = 2*arg1*arg4/(GB(arg1,arg2,arg3,arg4)+
sqrt(GB(arg1,arg2,arg3,arg4)^2-4*(arg2-arg1)*arg1*arg4))
```

```
# parameters
# low TM treatment: stress=5
# high TM treatment: stress=50
# low TG treatment: stress=0.5, TG=0.25
# high TG treatment: stress=50, TG=0.25
# low DTT treatment: stress=5, kapa=5
# high DTT treatment: stress=50, kapa=5
p stress=0, TG=0
p kbcs=10, kbcd=5, kbcd'=10, kbcd"=15
p kbaa=20, kbai=0.1, kbai'=20, kbai"=5, BaxT=1
p kaua=5, kaua'=2, kaui=0.5, kaui'=50, kaui"=5, AUTAT=1, kdiss=1, kass=100
p kapa=0.01, kapa'=10, kapa"=150, kapa"'=15, kapi=50, kapi'=100, APOT=1, Jap=0.01

done
```

2 Supplementary figure

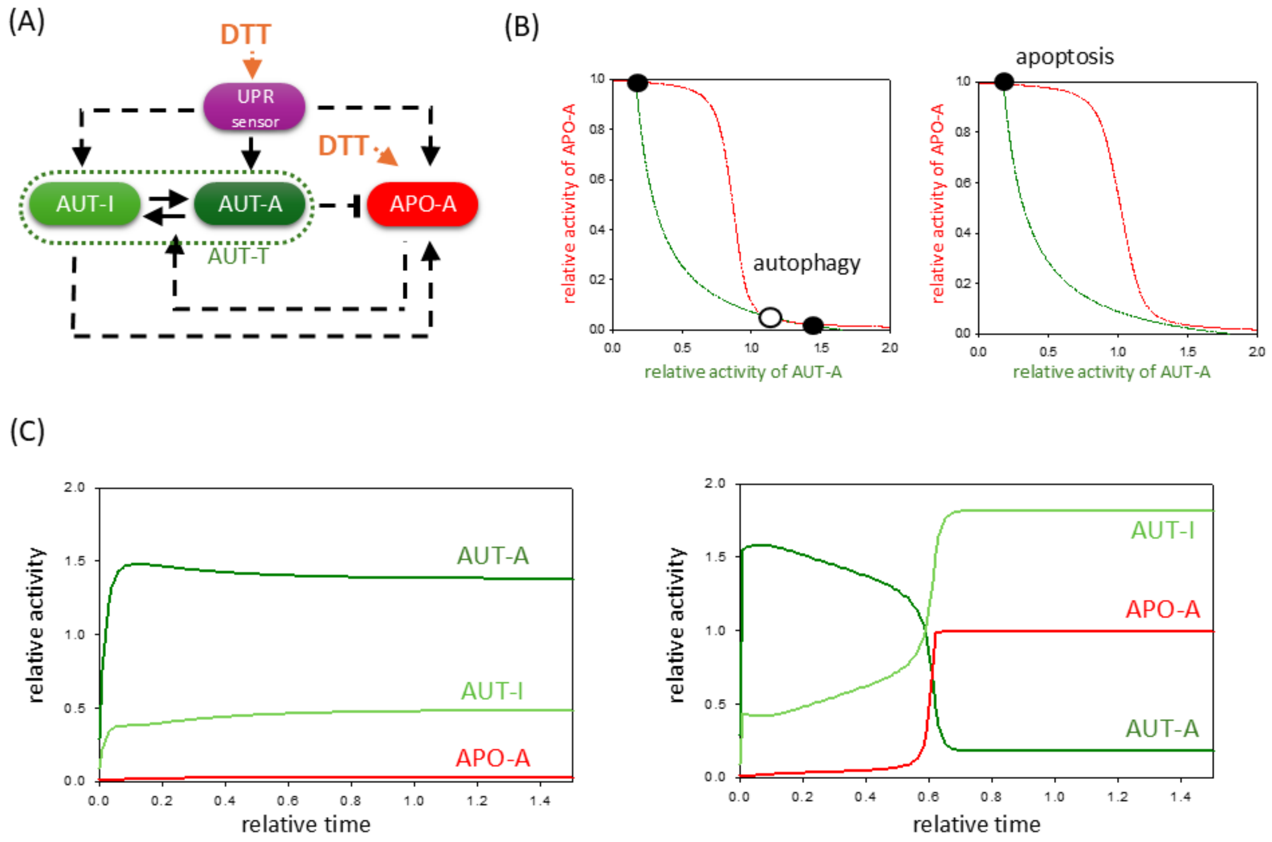

Figure S1: DTT-induced ER stress acts indirectly on autophagy and apoptosis pathways via UPR. (**A**) The wiring diagram of the control network with respect to ER stress. DTT indicates the effect of dithiotreitol. The UPR sensor, autophagy inducer (AUT-A), the apoptosis inducer (APO-A) are grouped together in isolated purple, green and red boxes, respectively. The active forms of the molecules are grouped in dark-coloured boxes, while light-coloured box denotes the inactive form. Solid arrows represent biochemical reactions, dashed line shows how the molecules can influence each other. Blocked end lines denote inhibition. (**B**) Phase plane diagrams are plotted upon (panel left) low (stress = 5) and (panel right) high (stress = 50) level of TM stress. The balance curves of AUT-A (green) and APO-A (red) are plotted. Intersection of nullclines represents the stable (black dots) and unstable (unfilled circle) steady states. (**C**) The temporal dynamics of AUT-A, APO-A and AUT-I are plotted upon low (panel left, stress = 5) and high (panel right, stress = 50) level of ER stress.

3 Supplementary table

Supplementary Table S1. Collection of data from literature on proven regulatory links in the regulatory network. Arrows indicate activation, while blocked end lines indicate inhibition between the members of the network.

Connection	Reference	Cell type/tissue/organism
**CHOP/JNK (UPR) -| BCL2**	Gross et al, 2017	review
	Yamamoto et al, 1999	Jurkat cell, WEHI-231
	McCullough et al, 2001	HeLa, Rat1, Rat-Myc, MEF
	Szegezdi et al, 2009	review
	Bassik et al, 2004	Fl 5.12 lines, Jurkat cell, MEF
	Rodriguez et al, 2011	review
	Puthalakath et al, 2007	mice, Thymocytes, peritoneal macrophages, MCF-7, MEF
	Oakes et al, 2006	review
	Wei et al, 2008	HeLa, MCF-7, MEF
	Kania et al, 2015	review
	Luo et al, 2013	review
	Høyer-Hansen et al, 2007	review
	Šrámek et al, 2021	review
	Sano et al, 2013	review
**BCL2 -| Beclin1 (AUT-A)**	Brahmbhatt et al, 2015	review
	Chang et al, 2010	SK-Mel5, H1299 neo, HA-BCL-2b5
	Rodriguez et al, 2011	review
	Ciechomska et al, 2009	HeLa, MEF
	Pattingre et al, 2005	HEK293, HeLa, MCF-7, HT-29, mice
	Kang et al, 2011	review
	Kania et al, 2015	review
	Høyer-Hansen et al, 2007	review
	Vicencio et al, 2009	HeLa, Rat-1, DT40
	Heath-Engel et al, 2008	review
	Pihán et al, 2017	review
	Siddiqui et al, 2015	review
	Yang et al, 2019	mathematical modell
	Maiuri et al, 2007	HeLa, MEF, Acta, Cb5
	Marquez et al, 2012	review
**BCL2-| AMBRA1 (AUT-A)**	Yang et al, 2019	mathematical modell
	Strappazzon et al, 2011	HEK293, HeLa
	Fimia et al, 2012	review
**inactive AMBRA1 (AUT-I) -> APO-A**	Strappazzon et al, 2016	HEK293, HeLa
**inactive Beclin1 (AUT-I) -> BAX**	Siddiqui et al, 2015	review
**BCL2 -| APO-A**	Brahmbhatt et al, 2015	review
	Rong et al, 2009	COS-7, WEHI7.2, Jurkat cell
	Šrámek et al, 2021	review
	Sano et al, 2013	review
	Heath-Engel et al, 2008	review
	Häcki et al, 2000	Rat 6, JILY B
	Yang et al, 2019	mathematical modell
**BCL2 -| BAX**	Šrámek et al, 2021	review
	Lindqvist et al, 2014	MEF, myeloid cell
	Szegezdi et al, 2006	review
	Bhatt et al, 2008	rat kidney proximal tubular cell line
	Yang et al, 2019	mathematical modell
	Marquez et al, 2012	review
**BAX -| BCL2**	Pihán et al, 2017	review
**BAX -> Casp9 (APO-A)**	Šrámek et al, 2021	review
	Lei et al, 2003	HEK293T
	Riedl et al, 2004	review
	Zong et al, 2003	murine fibroblasts, MEF, MCF-7, Phoenix cells
**Caspases (APO-A) -| BCL2**	Kirsch et al, 1999	SK-NSH, HL-60, MCF-7
	Zhu et al, 2007	CHO AA8, MCF-7 , BEL-7404
**inactive Beclin1 (AUT-I) -> Caspases (APO-A)**	Zhu et al, 2007	CHO AA8, MCF-7 , BEL-7404
	Wirawan et al, 2010	Ba/F3, FDCP1, U937, HeLa
	Zhu et al, 2010	HeLa
	Huang et al, 2014	U87, U251, Human astrocytes-cerebellar (HAc) astroglial cells
	Li et al, 2016	SKOV-3
	Gordy et al, 2012	review
**Caspases (APO-A) -I Beclin1 (AUT-A)**	Siddiqui et al, 2015	review
	Gordy et al, 2012	review
	Djavaheri-Mergny et al, 2010	review
	Wirawan et al, 2010	Ba/F3, FDCP1, U937, HeLa
	Luo et al, 2010	HeLa, SK-N-SH, MEF
**AUT-A -| Caspases (APO-A)**	Hou et al, 2010	Hct116, Jurkat cell
**Beclin1 (AUT-A) -| Caspases (APO-A)**	Ciechomska et al, 2009	HeLa, MEF
**BCL2 -I GABARAP (AUT-A)**	Ma et al, 2013	Rat embryonic fibroblast (REF), Jurkat cell, MEF
**GABARAP (AUT-A) -> APO-A**	Lee et al, 2005	Human ovarian tumor 2774 cell, SKOV-3

Supplementary Table S2. Collection of data from the literature on the effects of the compounds (tunicamycin, thapsigargin, DTT) on the regulatory network. These compounds directly activate the UPR, the apoptosis or the autophagy.

	UPR			APOA			AUTA		
	Cell type/tissue/organism	active protein		Cell type/tissue/organism	active protein		Cell type/tissue/organism	active protein	
TM	SGC7901, BGC823, MKN45, AGS, the human immortalized gastric epithelial cell line	PERK, IRE1, XBP1s	Wu et al, 2018						
	review		Banerjee et al, 2024						
	Rat chondrocytes	GRP78, ATF4, CHOP, GADD34	Wu et al, 2020						
	HN4, CAL27	PDI, IRE1α, GRP78, Ero1-Lα, calnexin	Wang et al, 2020						
	PC3	FOXO4, ATF3, CHOP	Guha et al, 2017						
	LLC-PK1, MEF, mice	GRP78, IRE1, PERK, eIF2α	Chandrika et al, 2015						
	LNCaP (CRL-1740), PC3 (CRL-1435), RPE-1, HeLaT	PERK, ATF4, IRE1, CHOP, XBP1s, GRP78	Luhr et al, 2019						
	Arabidopsis thaliana	IRE1b	Yang et al, 2015						
TG	review		Jaskulska et al, 2020	review		Jaskulska et al, 2020	HepG2	Beclin-1	Wang et al, 2016
	LNCaP, HCT116	PERK, ATF4, CHOP, IRE1, XBP1s, JNK, GRP78	Lindner et al, 2020	HepG2	increasing the Bax/Bcl-2 ratio	Wang et al, 2016	J16, DT40, MEF, U2OS	WIPI-1, LC3, Ca(2+)-mediated autophagy	Grotemeier et al, 2010
	SK-N-SH, MEF	IRE1	Ogata et al, 2006	LNCaP, PC3	Ca2+ depletion	Sehgal et al, 2017	MCF-7S1, HeLa, NIH3T3, MCF10A	CaMKK-β, AMPK	Høyer-Hansen et al, 2007
	Immortalized hepatocytes, Primary murine hepatocytes	CHOP, XBP1s	Sakaki et al, 2008	LNCaP, HCT116	DR5, caspase-8	Lindner et al, 2020	review		Rashid et al, 2015
				HCT116	DR5, caspase-8	Lam et al, 2018			
				HCT116, HeLa, MDA-MB-468, A2780S, HEK293, DU145, LNCaP	DR5	Yamaguchi et al, 2004			
				HCT116, KMS11, SK-MES-1	DR5, caspase-8	Lu et al, 2014			
				EC109, TE12, HET-1A	DR5, AMPK	Ma et al, 2016			
				review		Muñoz-Pinedo et al, 2018			
DTT	HeLa	GRP78, CHOP, JNK	Xiang et al, 2016	HL-60	caspase 3	Tartier et al, 2000			
	Arabidopsis thaliana	IRE1b	Yang et al, 2015	review		Held et al, 1996			
	HEK293, HeLa 229, mice	IRE1	Keestra-Gounder et al, 2016	V79	H2O2 is formed	Held et al, 1987			
	S. cerevisiae	Kar2p (GRP78)	Yorimitsu et al, 2007	Trypanosoma cruzi	rupture the mitochondrion homeostasis	Sandes et al, 2019			
	C. elegans	ire1, xbp1, atf6	Gokul et al, 2022	C. elegans	via the modulation of the methionine–homocysteine cycle	Gokul et al, 2022			
				C. elegans, HeLa, LN229, Cal27	cysl-1, rips-1 (hypoxia response pathway)	Ravi et al, 2023			

Supplementary Table S3. A summary of a comparison of the best known ER stressors based on their effects on autophagy and apoptosis.

	TG	TM	DTT
direct UPR induction	✓	✓	✓
direct AUT induction	✓	✗	✗
direct APO induction	✓	✗	✓
low concentration of stressor induces AUT followed by APO	✓	✗	✗
low concentration of stressor induces only AUT	✗	✓	✓
high concentration of stressor induces AUT followed by APO	✓	✓	✓
washable	✗	✓	✓
its effect delayable with AUT activator	✓	✓	✓
additive	✓	✓	✓

4 References

1. Banerjee, S.; Ansari, A.A.; Upadhyay, S.P.; Mettman, D.J.; Hibdon, J.R.; Quadir, M.; Ghosh, P.; Kambhampati, A.; Banerjee, S.K. Benefits and Pitfalls of a Glycosylation Inhibitor Tunicamycin in the Therapeutic Implication of Cancers. Cells 2024, 13, doi:10.3390/cells13050395.
2. Bassik, M.C.; Scorrano, L.; Oakes, S.A.; Pozzan, T.; Korsmeyer, S.J. Phosphorylation of BCL-2 regulates ER Ca2+ homeostasis and apoptosis. EMBO J 2004, 23, 1207-1216, doi:10.1038/sj.emboj.7600104.
3. Bhatt, K.; Feng, L.; Pabla, N.; Liu, K.; Smith, S.; Dong, Z. Effects of targeted Bcl-2 expression in mitochondria or endoplasmic reticulum on renal tubular cell apoptosis. Am J Physiol Renal Physiol 2008, 294, F499-507, doi:10.1152/ajprenal.00415.2007.
4. Brahmbhatt, H.; Oppermann, S.; Osterlund, E.J.; Leber, B.; Andrews, D.W. Molecular Pathways: Leveraging the BCL-2 Interactome to Kill Cancer Cells–Mitochondrial Outer Membrane Permeabilization and Beyond. Clin Cancer Res 2015, 21, 2671-2676, doi:10.1158/1078-0432.CCR-14-0959.
5. Chandrika, B.B.; Yang, C.; Ou, Y.; Feng, X.; Muhoza, D.; Holmes, A.F.; Theus, S.; Deshmukh, S.; Haun, R.S.; Kaushal, G.P. Endoplasmic Reticulum Stress-Induced Autophagy Provides Cytoprotection from Chemical Hypoxia and Oxidant Injury and Ameliorates Renal Ischemia-Reperfusion Injury. PLoS One 2015, 10, e0140025, doi:10.1371/journal.pone.0140025.
6. Chang, N.C.; Nguyen, M.; Germain, M.; Shore, G.C. Antagonism of Beclin 1-dependent autophagy by BCL-2 at the endoplasmic reticulum requires NAF-1. EMBO J 2010, 29, 606-618, doi:10.1038/emboj.2009.369.
7. Ciechomska, I.A.; Goemans, G.C.; Skepper, J.N.; Tolkovsky, A.M. Bcl-2 complexed with Beclin-1 maintains full anti-apoptotic function. Oncogene 2009, 28, 2128-2141, doi:10.1038/onc.2009.60.
8. Djavaheri-Mergny, M.; Maiuri M. C.; Kroemer G. Cross talk between apoptosis and autophagy by caspase-mediated cleavage of Beclin 1. Oncogene 2010, 29(12): 1717-1719.
doi: 10.1038/onc.2009.519
9. Fimia, G.M.; Corazzari, M.; Antonioli, M.; Piacentini, M. Ambra1 at the crossroad between autophagy and cell death. Oncogene 2013, 32, 3311-3318, doi:10.1038/onc.2012.455.
10. G, G.; Singh, J. Dithiothreitol causes toxicity in C. elegans by modulating the methionine-homocysteine cycle. Elife 2022, 11, doi:10.7554/eLife.76021.
11. Gordy, C.; He, Y.W. The crosstalk between autophagy and apoptosis: where does this lead? Protein Cell 2012, 3, 17-27, doi:10.1007/s13238-011-1127-x.
12. Gross, A.; Katz, S.G. Non-apoptotic functions of BCL-2 family proteins. Cell Death Differ 2017, 24, 1348-1358, doi:10.1038/cdd.2017.22.
13. Grotemeier, A.; Alers, S.; Pfisterer, S.G.; Paasch, F.; Daubrawa, M.; Dieterle, A.; Viollet, B.; Wesselborg, S.; Proikas-Cezanne, T.; Stork, B. AMPK-independent induction of autophagy by cytosolic Ca2+ increase. Cell Signal 2010, 22, 914-925, doi:10.1016/j.cellsig.2010.01.015.
14. Guha, P.; Kaptan, E.; Gade, P.; Kalvakolanu, D.V.; Ahmed, H. Tunicamycin induced endoplasmic reticulum stress promotes apoptosis of prostate cancer cells by activating mTORC1. Oncotarget 2017, 8, 68191-68207, doi:10.18632/oncotarget.19277.

15. Hacki, J.; Egger, L.; Monney, L.; Conus, S.; Rosse, T.; Fellay, I.; Borner, C. Apoptotic crosstalk between the endoplasmic reticulum and mitochondria controlled by Bcl-2. Oncogene 2000, 19, 2286-2295, doi:10.1038/sj.onc.1203592.
16. Heath-Engel, H.M.; Chang, N.C.; Shore, G.C. The endoplasmic reticulum in apoptosis and autophagy: role of the BCL-2 protein family. Oncogene 2008, 27, 6419-6433, doi:10.1038/onc.2008.309.
17. Held, K.D.; Melder, D.C. Toxicity of the sulfhydryl-containing radioprotector dithiothreitol. Radiat Res 1987, 112, 544-554. 18. Held, K.D.; Sylvester, F.C.; Hopcia, K.L.; Biaglow, J.E. Role of Fenton chemistry in thiol-induced toxicity and apoptosis. Radiat Res 1996, 145, 542-553.
19. Hou, W.; Han, J.; Lu, C.; Goldstein, L.A.; Rabinowich, H. Autophagic degradation of active caspase-8: a crosstalk mechanism between autophagy and apoptosis. Autophagy 2010, 6, 891-900, doi:10.4161/auto.6.7.13038.
20. Hoyer-Hansen, M.; Bastholm, L.; Szyniarowski, P.; Campanella, M.; Szabadkai, G.; Farkas, T.; Bianchi, K.; Fehrenbacher, N.; Elling, F.; Rizzuto, R.; et al. Control of macroautophagy by calcium, calmodulin-dependent kinase kinase-beta, and Bcl-2. Mol Cell 2007, 25, 193-205, doi:10.1016/j.molcel.2006.12.009.
21. Hoyer-Hansen, M.; Jaattela, M. Connecting endoplasmic reticulum stress to autophagy by unfolded protein response and calcium. Cell Death Differ 2007, 14, 1576-1582, doi:10.1038/sj.cdd.4402200.
22. Huang, X.; Qi, Q.; Hua, X.; Li, X.; Zhang, W.; Sun, H.; Li, S.; Wang, X.; Li, B. Beclin 1, an autophagy-related gene, augments apoptosis in U87 glioblastoma cells. Oncol Rep 2014, 31, 1761-1767, doi:10.3892/or.2014.3015.
23. Jaskulska, A.; Janecka, A.E.; Gach-Janczak, K. Thapsigargin-From Traditional Medicine to Anticancer Drug. Int J Mol Sci 2020, 22, doi:10.3390/ijms22010004.
24. Kang, R.; Zeh, H.J.; Lotze, M.T.; Tang, D. The Beclin 1 network regulates autophagy and apoptosis. Cell Death Differ 2011, 18, 571-580, doi:10.1038/cdd.2010.191.
25. Kania, E.; Pajak, B.; Orzechowski, A. Calcium homeostasis and ER stress in control of autophagy in cancer cells. Biomed Res Int 2015, 2015, 352794, doi:10.1155/2015/352794.
26. Keestra-Gounder, A.M.; Byndloss, M.X.; Seyffert, N.; Young, B.M.; Chavez-Arroyo, A.; Tsai, A.Y.; Cevallos, S.A.; Winter, M.G.; Pham, O.H.; Tiffany, C.R.; et al. NOD1 and NOD2 signalling links ER stress with inflammation. Nature 2016, 532, 394-397, doi:10.1038/nature17631.
27. Kirsch, D.G.; Doseff, A.; Chau, B.N.; Lim, D.S.; de Souza-Pinto, N.C.; Hansford, R.; Kastan, M.B.; Lazebnik, Y.A.; Hardwick, J.M. Caspase-3-dependent cleavage of Bcl-2 promotes release of cytochrome c. J Biol Chem 1999, 274, 21155-21161, doi:10.1074/jbc.274.30.21155.
28. Lam, M.; Lawrence, D.A.; Ashkenazi, A.; Walter, P. Confirming a critical role for death receptor 5 and caspase-8 in apoptosis induction by endoplasmic reticulum stress. Cell Death Differ 2018, 25, 1530-1531, doi:10.1038/s41418-018-0155-y.
29. Lee, J.H.; Rho, S.B.; Chun, T. GABAA receptor-associated protein (GABARAP) induces apoptosis by interacting with DEAD (Asp-Glu-Ala-Asp/His) box polypeptide 47 (DDX 47). Biotechnol Lett 2005, 27, 623-628, doi:10.1007/s10529-005-3628-2.
30. Lei, K.; Davis, R.J. JNK phosphorylation of Bim-related members of the Bcl2 family induces Bax-dependent apoptosis. Proc Natl Acad Sci U S A 2003, 100, 2432-2437, doi:10.1073/pnas.0438011100.

31. Li, X.; Su, J.; Xia, M.; Li, H.; Xu, Y.; Ma, C.; Ma, L.; Kang, J.; Yu, H.; Zhang, Z.; et al. Caspase-mediated cleavage of Beclin1 inhibits autophagy and promotes apoptosis induced by S1 in human ovarian cancer SKOV3 cells. Apoptosis 2016, 21, 225-238, doi:10.1007/s10495-015-1197-y.
32. Lindner, P.; Christensen, S.B.; Nissen, P.; Moller, J.V.; Engedal, N. Cell death induced by the ER stressor thapsigargin involves death receptor 5, a non-autophagic function of MAP1LC3B, and distinct contributions from unfolded protein response components. Cell Commun Signal 2020, 18, 12, doi:10.1186/s12964-019-0499-z.
33. Lindqvist, L.M.; Heinlein, M.; Huang, D.C.; Vaux, D.L. Prosurvival Bcl-2 family members affect autophagy only indirectly, by inhibiting Bax and Bak. Proc Natl Acad Sci U S A 2014, 111, 8512-8517, doi:10.1073/pnas.1406425111.
34. Lu, M.; Lawrence, D.A.; Marsters, S.; Acosta-Alvear, D.; Kimmig, P.; Mendez, A.S.; Paton, A.W.; Paton, J.C.; Walter, P.; Ashkenazi, A. Opposing unfolded-protein-response signals converge on death receptor 5 to control apoptosis. Science 2014, 345, 98-101, doi:10.1126/science.1254312.
35. Luhr, M.; Torgersen, M.L.; Szalai, P.; Hashim, A.; Brech, A.; Staerk, J.; Engedal, N. The kinase PERK and the transcription factor ATF4 play distinct and essential roles in autophagy resulting from tunicamycin-induced ER stress. J Biol Chem 2019, 294, 8197-8217, doi:10.1074/jbc.RA118.002829.
36. Luo, B.; Lee, A.S. The critical roles of endoplasmic reticulum chaperones and unfolded protein response in tumorigenesis and anticancer therapies. Oncogene 2013, 32, 805-818, doi:10.1038/onc.2012.130.
37. Luo, S.; Rubinsztein, D.C. Apoptosis blocks Beclin 1-dependent autophagosome synthesis: an effect rescued by Bcl-xL. Cell Death Differ 2010, 17, 268-277, doi:10.1038/cdd.2009.121.
38. Ma, P.; Schwarten, M.; Schneider, L.; Boeske, A.; Henke, N.; Lisak, D.; Weber, S.; Mohrluder, J.; Stoldt, M.; Strodel, B.; et al. Interaction of Bcl-2 with the autophagy-related GABAA receptor-associated protein (GABARAP): biophysical characterization and functional implications. J Biol Chem 2013, 288, 37204-37215, doi:10.1074/jbc.M113.528067.
39. Ma, Z.; Fan, C.; Yang, Y.; Di, S.; Hu, W.; Li, T.; Zhu, Y.; Han, J.; Xin, Z.; Wu, G.; et al. Thapsigargin sensitizes human esophageal cancer to TRAIL-induced apoptosis via AMPK activation. Sci Rep 2016, 6, 35196, doi:10.1038/srep35196.
40. Maiuri, M.C.; Le Toumelin, G.; Criollo, A.; Rain, J.C.; Gautier, F.; Juin, P.; Tasdemir, E.; Pierron, G.; Troulinaki, K.; Tavernarakis, N.; et al. Functional and physical interaction between Bcl-X(L) and a BH3-like domain in Beclin-1. EMBO J 2007, 26, 2527-2539, doi:10.1038/sj.emboj.7601689.
41. Marquez, R.T.; Xu, L. Bcl-2:Beclin 1 complex: multiple, mechanisms regulating autophagy/apoptosis toggle switch. Am J Cancer Res 2012, 2, 214-221.
42. McCullough, K.D.; Martindale, J.L.; Klotz, L.O.; Aw, T.Y.; Holbrook, N.J. Gadd153 sensitizes cells to endoplasmic reticulum stress by down-regulating Bcl2 and perturbing the cellular redox state. Mol Cell Biol 2001, 21, 1249-1259, doi:10.1128/MCB.21.4.1249-1259.2001.
43. Munoz-Pinedo, C.; Lopez-Rivas, A. A role for caspase-8 and TRAIL-R2/DR5 in ER-stress-induced apoptosis. Cell Death Differ 2018, 25, 226, doi:10.1038/cdd.2017.155.
44. Oakes, S.A.; Lin, S.S.; Bassik, M.C. The control of endoplasmic reticulum-initiated apoptosis by the BCL-2 family of proteins. Curr Mol Med 2006, 6, 99-109,

doi:10.2174/156652406775574587.
45. Ogata, M.; Hino, S.; Saito, A.; Morikawa, K.; Kondo, S.; Kanemoto, S.; Murakami, T.; Taniguchi, M.; Tanii, I.; Yoshinaga, K.; et al. Autophagy is activated for cell survival after endoplasmic reticulum stress. Mol Cell Biol 2006, 26, 9220-9231, doi:10.1128/MCB.01453-06.
46. Pattingre, S.; Tassa, A.; Qu, X.; Garuti, R.; Liang, X.H.; Mizushima, N.; Packer, M.; Schneider, M.D.; Levine, B. Bcl-2 antiapoptotic proteins inhibit Beclin 1-dependent autophagy. Cell 2005, 122, 927-939, doi:10.1016/j.cell.2005.07.002.
47. Pihan, P.; Carreras-Sureda, A.; Hetz, C. BCL-2 family: integrating stress responses at the ER to control cell demise. Cell Death Differ 2017, 24, 1478-1487, doi:10.1038/cdd.2017.82.
48. Puthalakath, H.; O'Reilly, L.A.; Gunn, P.; Lee, L.; Kelly, P.N.; Huntington, N.D.; Hughes, P.D.; Michalak, E.M.; McKimm-Breschkin, J.; Motoyama, N.; et al. ER stress triggers apoptosis by activating BH3-only protein Bim. Cell 2007, 129, 1337-1349, doi:10.1016/j.cell.2007.04.027.
49. Rashid, H.O.; Yadav, R.K.; Kim, H.R.; Chae, H.J. ER stress: Autophagy induction, inhibition and selection. Autophagy 2015, 11, 1956-1977, doi:10.1080/15548627.2015.1091141.
50. Ravi; Kumar, A.; Bhattacharyya, S.; Singh, J. Thiol reductive stress activates the hypoxia response pathway. EMBO J 2023, 42, e114093, doi:10.15252/embj.2023114093.
51. Riedl, S.J.; Shi, Y. Molecular mechanisms of caspase regulation during apoptosis. Nat Rev Mol Cell Biol 2004, 5, 897-907, doi:10.1038/nrm1496.
52. Rodriguez, D.; Rojas-Rivera, D.; Hetz, C. Integrating stress signals at the endoplasmic reticulum: The BCL-2 protein family rheostat. Biochim Biophys Acta 2011, 1813, 564-574, doi:10.1016/j.bbamcr.2010.11.012.
53. Rong, Y.P.; Bultynck, G.; Aromolaran, A.S.; Zhong, F.; Parys, J.B.; De Smedt, H.; Mignery, G.A.; Roderick, H.L.; Bootman, M.D.; Distelhorst, C.W. The BH4 domain of Bcl-2 inhibits ER calcium release and apoptosis by binding the regulatory and coupling domain of the IP3 receptor. Proc Natl Acad Sci U S A 2009, 106, 14397-14402, doi:10.1073/pnas.0907555106.
54. Sakaki, K.; Wu, J.; Kaufman, R.J. Protein kinase Ctheta is required for autophagy in response to stress in the endoplasmic reticulum. J Biol Chem 2008, 283, 15370-15380, doi:10.1074/jbc.M710209200.
55. Sano, R.; Reed, J.C. ER stress-induced cell death mechanisms. Biochim Biophys Acta 2013, 1833, 3460-3470, doi:10.1016/j.bbamcr.2013.06.028.
56. Sehgal, P.; Szalai, P.; Olesen, C.; Praetorius, H.A.; Nissen, P.; Christensen, S.B.; Engedal, N.; Moller, J.V. Inhibition of the sarco/endoplasmic reticulum (ER) Ca(2+)-ATPase by thapsigargin analogs induces cell death via ER Ca(2+) depletion and the unfolded protein response. J Biol Chem 2017, 292, 19656-19673, doi:10.1074/jbc.M117.796920.
57. Siddiqui, M.A.; Mukherjee, S.; Manivannan, P.; Malathi, K. RNase L Cleavage Products Promote Switch from Autophagy to Apoptosis by Caspase-Mediated Cleavage of Beclin-1. Int J Mol Sci 2015, 16, 17611-17636, doi:10.3390/ijms160817611.
58. Siddiqui, W.A.; Ahad, A.; Ahsan, H. The mystery of BCL2 family: Bcl-2 proteins and apoptosis: an update. Arch Toxicol 2015, 89, 289-317, doi:10.1007/s00204-014-1448-7.
59. Sramek, J.; Nemcova-Furstova, V.; Kovar, J. Molecular Mechanisms of Apopto-

sis Induction and Its Regulation by Fatty Acids in Pancreatic beta-Cells. Int J Mol Sci 2021, 22, doi:10.3390/ijms22084285.
60. Strappazzon, F.; Di Rita, A.; Cianfanelli, V.; D'Orazio, M.; Nazio, F.; Fimia, G.M.; Cecconi, F. Prosurvival AMBRA1 turns into a proapoptotic BH3-like protein during mitochondrial apoptosis. Autophagy 2016, 12, 963-975, doi:10.1080/15548627.2016.1164359.
61. Strappazzon, F.; Vietri-Rudan, M.; Campello, S.; Nazio, F.; Florenzano, F.; Fimia, G.M.; Piacentini, M.; Levine, B.; Cecconi, F. Mitochondrial BCL-2 inhibits AMBRA1-induced autophagy. EMBO J 2011, 30, 1195-1208, doi:10.1038/emboj.2011.49.
62. Szegezdi, E.; Logue, S.E.; Gorman, A.M.; Samali, A. Mediators of endoplasmic reticulum stress-induced apoptosis. EMBO Rep 2006, 7, 880-885, doi:10.1038/sj.embor.7400779.
63. Szegezdi, E.; Macdonald, D.C.; Ni Chonghaile, T.; Gupta, S.; Samali, A. Bcl-2 family on guard at the ER. Am J Physiol Cell Physiol 2009, 296, C941-953, doi:10.1152/ajpcell.00612.2008.
64. Tartier, L.; McCarey, Y.L.; Biaglow, J.E.; Kochevar, I.E.; Held, K.D. Apoptosis induced by dithiothreitol in HL-60 cells shows early activation of caspase 3 and is independent of mitochondria. Cell Death Differ 2000, 7, 1002-1010, doi:10.1038/sj.cdd.4400726.
65. Vicencio, J.M.; Ortiz, C.; Criollo, A.; Jones, A.W.; Kepp, O.; Galluzzi, L.; Joza, N.; Vitale, I.; Morselli, E.; Tailler, M.; et al. The inositol 1,4,5-trisphosphate receptor regulates autophagy through its interaction with Beclin 1. Cell Death Differ 2009, 16, 1006-1017, doi:10.1038/cdd.2009.34.
66. Wang, C.; Li, T.; Tang, S.; Zhao, D.; Zhang, C.; Zhang, S.; Deng, S.; Zhou, Y.; Xiao, X. Thapsigargin induces apoptosis when autophagy is inhibited in HepG2 cells and both processes are regulated by ROS-dependent pathway. Environ Toxicol Pharmacol 2016, 41, 167-179, doi:10.1016/j.etap.2015.11.020.
67. Wang, Y.; Zhang, L.; He, Z.; Deng, J.; Zhang, Z.; Liu, L.; Ye, W.; Liu, S. Tunicamycin induces ER stress and inhibits tumorigenesis of head and neck cancer cells by inhibiting N-glycosylation. Am J Transl Res 2020, 12, 541-550.
68. Wei, Y.; Pattingre, S.; Sinha, S.; Bassik, M.; Levine, B. JNK1-mediated phosphorylation of Bcl-2 regulates starvation-induced autophagy. Mol Cell 2008, 30, 678-688, doi:10.1016/j.molcel.2008.06.001.
69. Wirawan, E.; Vande Walle, L.; Kersse, K.; Cornelis, S.; Claerhout, S.; Vanoverberghe, I.; Roelandt, R.; De Rycke, R.; Verspurten, J.; Declercq, W.; et al. Caspase-mediated cleavage of Beclin-1 inactivates Beclin-1-induced autophagy and enhances apoptosis by promoting the release of proapoptotic factors from mitochondria. Cell Death Dis 2010, 1, e18, doi:10.1038/cddis.2009.16.
70. Wu, H.; Meng, Z.; Jiao, Y.; Ren, Y.; Yang, X.; Liu, H.; Wang, R.; Cui, Y.; Pan, L.; Cao, Y. The endoplasmic reticulum stress induced by tunicamycin affects the viability and autophagy activity of chondrocytes. J Clin Lab Anal 2020, 34, e23437, doi:10.1002/jcla.23437.
71. Wu, J.; Chen, S.; Liu, H.; Zhang, Z.; Ni, Z.; Chen, J.; Yang, Z.; Nie, Y.; Fan, D. Tunicamycin specifically aggravates ER stress and overcomes chemoresistance in multidrug-resistant gastric cancer cells by inhibiting N-glycosylation. J Exp Clin Cancer Res 2018, 37, 272, doi:10.1186/s13046-018-0935-8.
72. Xiang, X.Y.; Yang, X.C.; Su, J.; Kang, J.S.; Wu, Y.; Xue, Y.N.; Dong,

Y.T.; Sun, L.K. Inhibition of autophagic flux by ROS promotes apoptosis during DTT-induced ER/oxidative stress in HeLa cells. Oncol Rep 2016, 35, 3471-3479, doi:10.3892/or.2016.4725.
73. Yamaguchi, H.; Wang, H.G. CHOP is involved in endoplasmic reticulum stress-induced apoptosis by enhancing DR5 expression in human carcinoma cells. J Biol Chem 2004, 279, 45495-45502, doi:10.1074/jbc.M406933200.
74. Yamamoto, K.; Ichijo, H.; Korsmeyer, S.J. BCL-2 is phosphorylated and inactivated by an ASK1/Jun N-terminal protein kinase pathway normally activated at G(2)/M. Mol Cell Biol 1999, 19, 8469-8478, doi:10.1128/MCB.19.12.8469.
75. Yang, B.; Liu, Q.; Bi, Y. Autophagy and apoptosis are regulated by stress on Bcl2 by AMBRA1 in the endoplasmic reticulum and mitochondria. Theor Biol Med Model 2019, 16, 18, doi:10.1186/s12976-019-0113-5.
76. Yang, X.; Srivastava, R.; Howell, S.H.; Bassham, D.C. Activation of autophagy by unfolded proteins during endoplasmic reticulum stress. Plant J 2016, 85, 83-95, doi:10.1111/tpj.13091.
77. Yorimitsu, T.; Nair, U.; Yang, Z.; Klionsky, D.J. Endoplasmic reticulum stress triggers autophagy. J Biol Chem 2006, 281, 30299-30304, doi:10.1074/jbc.M607007200.
78. Zhu, J.; Yang, Y.; Wu, J. Bcl-2 cleavages at two adjacent sites by different caspases promote cisplatin-induced apoptosis. Cell Res 2007, 17, 441-448, doi:10.1038/cr.2007.36.
79. Zhu, Y.; Zhao, L.; Liu, L.; Gao, P.; Tian, W.; Wang, X.; Jin, H.; Xu, H.; Chen, Q. Beclin 1 cleavage by caspase-3 inactivates autophagy and promotes apoptosis. Protein Cell 2010, 1, 468-477, doi:10.1007/s13238-010-0048-4.
80. Zong, W.X.; Li, C.; Hatzivassiliou, G.; Lindsten, T.; Yu, Q.C.; Yuan, J.; Thompson, C.B. Bax and Bak can localize to the endoplasmic reticulum to initiate apoptosis. J Cell Biol 2003, 162, 59-69, doi:10.1083/jcb.200302084.
